# Evolution of RNA-Protein Interactions: Non-Specific Binding Led to RNA Splicing Activity of Fungal Mitochondrial Tyrosyl-tRNA Synthetases

**DOI:** 10.1371/journal.pbio.1002028

**Published:** 2014-12-23

**Authors:** Lilian T. Lamech, Anna L. Mallam, Alan M. Lambowitz

**Affiliations:** The Institute for Cellular and Molecular Biology and Department of Molecular Biosciences, The University of Texas at Austin, Austin, Texas, United States of America; Stanford University, United States of America

## Abstract

The *Neurospora crassa* mitochondrial tyrosyl-tRNA synthetase (mtTyrRS; CYT-18 protein) evolved a new function as a group I intron splicing factor by acquiring the ability to bind group I intron RNAs and stabilize their catalytically active RNA structure. Previous studies showed: (i) CYT-18 binds group I introns by using both its N-terminal catalytic domain and flexibly attached C-terminal anticodon-binding domain (CTD); and (ii) the catalytic domain binds group I introns specifically via multiple structural adaptations that occurred during or after the divergence of Peziomycotina and Saccharomycotina. However, the function of the CTD and how it contributed to the evolution of splicing activity have been unclear. Here, small angle X-ray scattering analysis of CYT-18 shows that both CTDs of the homodimeric protein extend outward from the catalytic domain, but move inward to bind opposite ends of a group I intron RNA. Biochemical assays show that the isolated CTD of CYT-18 binds RNAs non-specifically, possibly contributing to its interaction with the structurally different ends of the intron RNA. Finally, we find that the yeast mtTyrRS, which diverged from Pezizomycotina fungal mtTyrRSs prior to the evolution of splicing activity, binds group I intron and other RNAs non-specifically via its CTD, but lacks further adaptations needed for group I intron splicing. Our results suggest a scenario of constructive neutral (i.e., pre-adaptive) evolution in which an initial non-specific interaction between the CTD of an ancestral fungal mtTyrRS and a self-splicing group I intron was “fixed” by an intron RNA mutation that resulted in protein-dependent splicing. Once fixed, this interaction could be elaborated by further adaptive mutations in both the catalytic domain and CTD that enabled specific binding of group I introns. Our results highlight a role for non-specific RNA binding in the evolution of RNA-binding proteins.

## Abstract 2

Studies of tRNA synthetases that adapted to assist the splicing of group I introns provide insight into how proteins can evolve new RNA-binding functions.

## Author Summary

The acquisition of new modes of post-transcriptional gene regulation played an important role in the evolution of eukaryotes and was achieved by an increase in the number of RNA-binding proteins with new functions. RNA-binding proteins bind directly to double- or single-stranded RNA and regulate many cellular processes. Here, we address how proteins evolve new RNA-binding functions by using as a model system a fungal mitochondrial tyrosyl-tRNA synthetase that evolved to acquire a novel function in splicing group I introns. Group I introns are RNA enzymes (or “ribozymes”) that catalyze their own removal from transcripts, but can become dependent upon proteins to stabilize their active structure. We show that the C-terminal domain of the synthetase is flexibly attached and has high non-specific RNA-binding activity that likely pre-dated the evolution of splicing activity. Our findings suggest an evolutionary scenario in which an initial non-specific interaction between an ancestral synthetase and a self-splicing group I intron was fixed by an intron RNA mutation, thereby making it dependent upon the protein for structural stabilization. The interaction then evolved by the acquisition of adaptive mutations throughout the protein and RNA that increased both the splicing efficiency and its protein-dependence. Our results suggest a general mechanism by which non-specific binding interactions can lead to the evolution of new RNA-binding functions and provide novel insights into splicing and synthetase mechanisms.

## Introduction

RNA-binding proteins play critical roles in post-transcriptional regulation of gene expression in all domains of life [1]. However, the complexity of this regulation is far greater in eukaryotes than in prokaryotes, reflecting both the larger number of RNAs requiring regulation and the evolution of new RNA processing and regulatory mechanisms. The latter include extensive RNA splicing and alternative splicing to produce different protein isoforms; an increased importance of RNA localization in larger and more complex eukaryotic cells; nonsense-mediated decay to prevent translation of intron-containing RNAs; and combinatorial regulation of mRNA translation and stability by RNA-binding proteins and miRNAs acting in ribonucleoprotein complexes [2]–[5]. These new modes of post-transcriptional regulation necessitated and were enabled by corresponding increases in the number and diversity of RNA-binding proteins and the evolution of new RNA-binding functions [3],[6]. Thus far, however, the molecular mechanisms underlying the evolution of new RNA-binding functions have remained unclear.

Cellular proteins that adapted to splice autocatalytic group I and group II introns provide powerful model systems for investigating how proteins evolve new RNA-binding functions. Group I and group II introns are found in prokaryotes and in the mitochondrial (mt) and chloroplast DNAs of some eukaryotes, with group I introns also found in the nuclear rRNA genes of certain fungi and protozoa [7],[8]. Both types of introns are ribozymes that catalyze their own splicing as well as mobile genetic elements that can be horizontally transferred to different hosts where they propagate by inserting into new genomic sites [9],[10]. Although some group I and II introns self-splice *in vitro*, most have acquired mutations that impair formation of a catalytically active RNA structure, necessitating the recruitment of cellular proteins to promote RNA folding for efficient splicing *in vivo* [11],[12]. These group I and group II intron splicing factors include both host-encoded proteins, such as aminoacyl-tRNA synthetases (aaRSs) and translation factors, and intron-encoded proteins, such as DNA endonuclease and reverse transcriptases, that evolved secondarily to function in RNA splicing [11]. Such co-option of pre-existing proteins to function in splicing is pertinent to the evolution of splicing mechanisms in higher organisms, as emphasized by recent findings that a key spliceosomal protein, Prp8, was derived from a group II intron-like reverse transcriptase [13],[14].

One of the most extensively studied examples of a cellular protein that evolved to function in RNA splicing is the *Neurospora crassa* mtTyrRS (CYT-18 protein), which acts as a splicing factor for mt group I introns [15]–[18]. Biochemical and structural studies showed that CYT-18 functions in splicing by recognizing and stabilizing the conserved phosphodiester backbone structure of group I intron RNAs [19]–[22]. This splicing function has been found only for those mtTyrRS of fungi belonging to the subphylum Pezizomycotina and can be traced to a series of structural adaptations of the protein that were acquired during or after the divergence of Pezizomycotina from Saccharomycotina [23].

**Figure 1:**
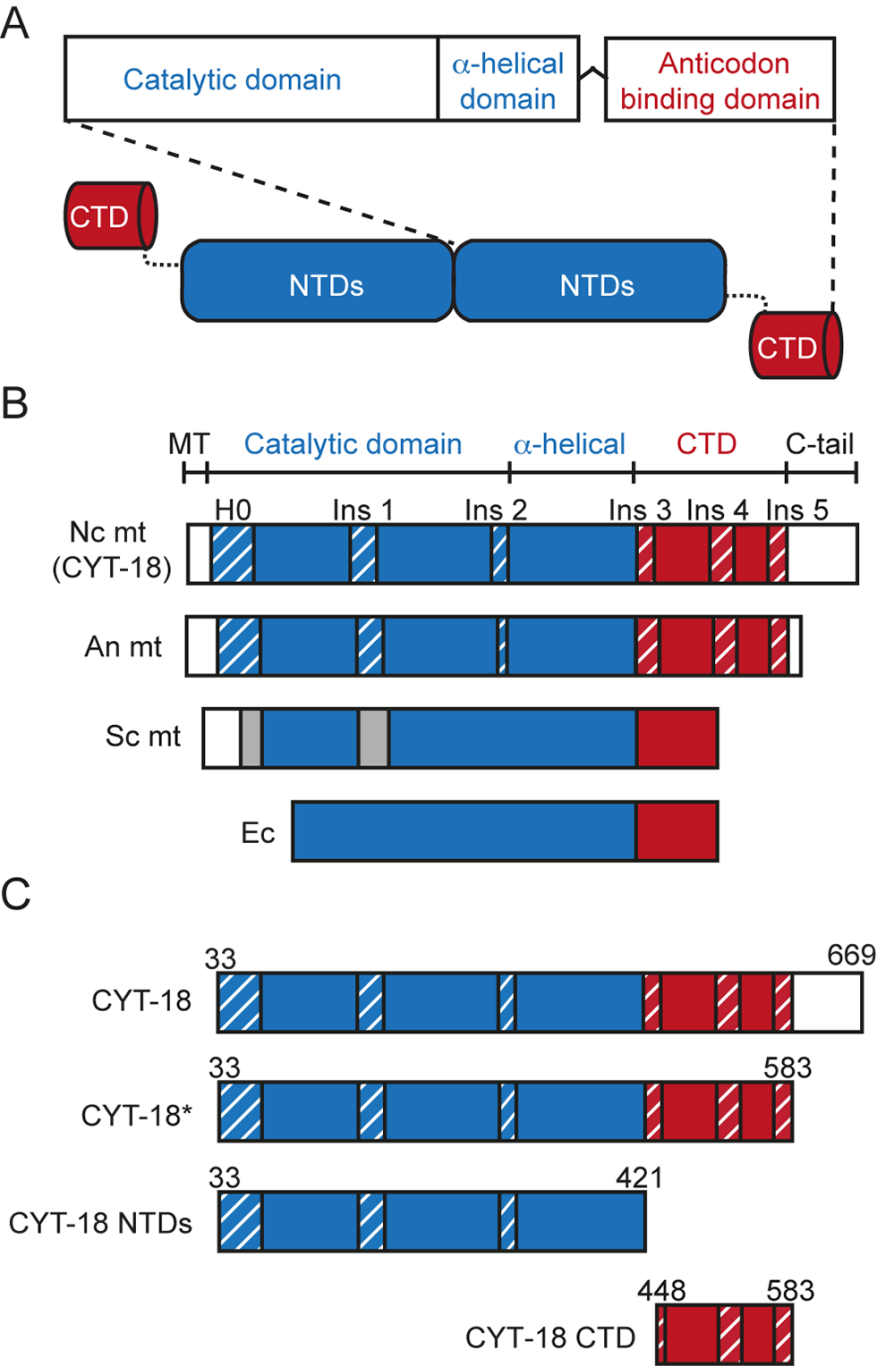
Domain architecture of the wild-type *N. crassa* mtTyrRS (CYT-18 protein) and deletion constructs. (A) Schematic of a CYT-18 homodimer. CYT-18 consists of N-terminal catalytic and α-helical domains (NTDs, blue) that are connected via a flexible linker to a CTD (red). The protein dimerizes via interactions between the catalytic domains. (B) Domain architecture of the *N. crassa* (Nc), *A. nidulans* (An), and *S. cerevisiae* (Sc) mtTyrRS compared to *E. coli* (Ec) TyrRS. The mitochondrial targeting sequence (MT) and C-terminal extension (C-tail) are in white, and Pezizomycotina-specific insertions in the NTD and CTD are highlighted with blue and red diagonal stripes, respectively. Insertions in the Sc mtTyrRS relative to *E. coli* TyrRS are indicated in gray and are not homologous to the Pezizomycotina insertions [23]. (C) CYT-18 protein constructs used in this study. The wild-type CYT-18 protein corresponds to the full-length mature protein lacking the mitochondrial targeting sequence. CYT-18* additionally lacks most of the non-essential C-tail and ends nine residues after Ins 5 (residue 583) to match the short C-tail of the *A. nidulans* mtTyrRS for which an NMR structure was determined [37]. CYT-18 NTDs contains the N-terminal catalytic and α-helical domains and lacks both the CTD and C-tail. The CTD construct begins with a few residues of the flexible linker (Ins 3) and ends at the same position as CYT-18*.

CYT-18 and other mtTyrRSs are class 1 aaRSs that are closely related to bacterial TyrRSs [24]. They consist of an N-terminal catalytic domain, which binds the acceptor stem of tRNA^Tyr^, followed by an intermediate α-helical domain and a C-terminal anticodon-binding domain (CTD), which bind the anticodon and variable arms (Figure 1A and 1B; the catalytic and intermediate α-helical domains together are denoted the N-terminal domains or NTDs). Like its bacterial counterparts, CYT-18 functions as a homodimer, with each dimer binding either one molecule of tRNA^Tyr^ or group I intron RNA [25]–[28]. CYT-18 binds group I introns by using both its N-terminal catalytic domain and CTD, but only some introns require the CTD for RNA splicing [29]–[31].

Both the N-terminal catalytic domain and CTD of Pezizomycotinia mtTyrRSs have distinctive structural adaptations that are absent in non-splicing mtTyrRSs, including the closely related Saccharomycotina mtTyrRSs [23]. These structural adaptations include a small N-terminal α-helical extension (denoted H0) and a series of small insertions (Ins 1–5), whose presence correlates with RNA splicing activity (Figure 1B) [32],[33]. The Pezizomycotina mtTyrRS also have a non-essential C-terminal tail of variable length (C-tail; 13–152 amino acids) appended to the CTD ([29] and this work).

Structural studies, including a co-crystal structure of a splicing-active CYT-18 protein lacking the CTD (here denoted CYT-18 NTDs) bound to a group I intron RNA (the bacteriophage Twort *orf*142-I2 ribozyme), provided insight into group I intron binding by the N-terminal catalytic domain [22],[33]. These studies showed that CYT-18 binds group I introns asymmetrically across the two subunits of the homodimer by using a newly evolved group I intron-binding surface on the side of the catalytic domain opposite that which binds tRNA^Tyr^. This new RNA-binding surface includes the N-terminal extension H0, Ins 1, and Ins 2 and provides an extended scaffold for the conserved phosphodiester backbone structure of the group I intron catalytic core.

The CYT-18 constructs used for crystallography lacked the flexibly attached CTD, which has been problematic for X-ray crystallography of TyrRSs [33]–[36]. Recently, we determined an NMR structure of the isolated CTD of the splicing-active *Aspergillus nidulans* mtTyrRS, which is closely related to CYT-18 [37]. The structure showed that the mtTyrRS CTD resembles those of bacterial TyrRSs in having a fold similar to that of bacterial ribosomal protein S4, but with novel structural features. The latter include three Pezizomycotina-specific insertions (Ins 3–5), with Ins 3 corresponding to an expansion of the flexible linker between the NTDs and CTD. Modeling of the NMR structure onto the CYT-18 NTDs+Twort co-crystal structure using distance constraints from directed hydroxyl-radical cleavage assays suggested that the two CTDs of the homodimeric protein bind opposite ends of a group I intron RNA. This model requires that the CTD of one subunit of the CYT-18 homodimer undergo a large shift on its flexible linker to interact with either tRNA^Tyr^ or the group I intron RNA bound on opposite sides of the catalytic domain [37]. Thus far, however, there has been no structural data for a CYT-18 protein that contains both the NTDs and CTD, and the role of the CTD in promoting group I intron splicing has remained unclear.

CYT-18 has been used as a model for the theory of constructive neutral evolution (referred to here as “pre-adaptive evolution”). This theory holds that complex multi-protein or RNP complexes arise by a “ratchet-like process” in which a pre-existing neutral or mildly deleterious interaction is “fixed” by a mutation in one partner that makes it dependent upon the other to perform a biological function. Once fixed, this dependence can be further elaborated by adaptive changes in both partners, which increase reaction efficiency and co-dependence [38]–[40]. In the case of CYT-18, this hypothesis suggests that an ancestral non-splicing fungal mtTyrRS had a pre-existing ability to bind group I introns, which became fixed when the intron RNA acquired mutations that impaired self-splicing, resulting in dependence upon the bound protein for structural stabilization [11]. After the interaction was fixed, further adaptive mutations in both the RNA and protein increased both the efficiency of RNA splicing and its protein-dependence. Early studies suggesting that CYT-18 recognized tRNA-like structural features of group I intron RNAs were cited as a prime example of a pre-adaptive interaction leading to the evolution of a new RNA-splicing function [39],[41]. However, subsequent findings that CYT-18's N-terminal catalytic domain binds group I introns specifically by using a separate non-tRNA-binding surface [22],[33] made the nature of the initial non-adaptive interaction unclear.

Here, we used small angle X-ray scattering (SAXS) and biofchemical assays to investigate the solution structures of full-length CYT-18 protein and its CTDs and their mode of interaction with group I intron RNAs. The SAXS analysis shows that the CTDs of both subunits of the CYT-18 homodimer extend outward from the NTDs, but move inward to bind opposite ends of the group I intron RNA. Surprisingly, we find that the CTD of CYT-18 has a high non-specific RNA binding affinity, which may contribute to its interaction with group I intron RNAs, and that the *Saccharomyces cerevisiae* (yeast) mtTyrRS, which diverged prior to the evolution of splicing activity, can also bind intron RNAs non-specifically via its CTD. Finally, experiments with chimeric proteins show that the yeast CTD can replace CYT-18's to promote aminoacylation but not group I intron splicing. Our results suggest a scenario of pre-adaptive evolution in which the initial non-adaptive interaction between an ancestral mtTyrRS and group I intron RNA was non-specific binding by the CTD and highlight a role for non-specific binding in the evolution of new RNA-binding functions.

## Results

### SAXS Analysis of CYT-18 and Its CTD

First, we used SAXS to investigate the conformational changes of CYT-18 and the position of its CTDs in the absence and presence of a group I intron RNA. Scattering data were collected for three CYT-18 constructs: CYT-18*, a wild-type protein truncated to delete most of the non-essential C-tail in order to simplify modeling and analysis; CYT-18 NTDs, which contains the N-terminal catalytic and α-helical domains, but lacks both the CTD and C-tail; and CTD, the isolated C-terminal anticodon-binding domain (Figure 1C). CYT-18* is fully active in tyrosyl-adenylation, which measures the number of TyrRS active sites, and it functions similarly to full-length CYT-18 both in aminoacylation of *Escherichia coli* tRNA^Tyr^, a standard substrate for this protein, and in splicing the *N. crassa* mt large subunit rRNA (Nc mt LSU) intron, which requires a functional CTD (Figure S1). The CYT-18 NTDs construct is also fully active in tyrosyl-adenylation, but cannot aminoacylate tRNA^Tyr^ as expected because of the lack of the CTD (Figure S1A and S1B) [30].

**Figure 2:**
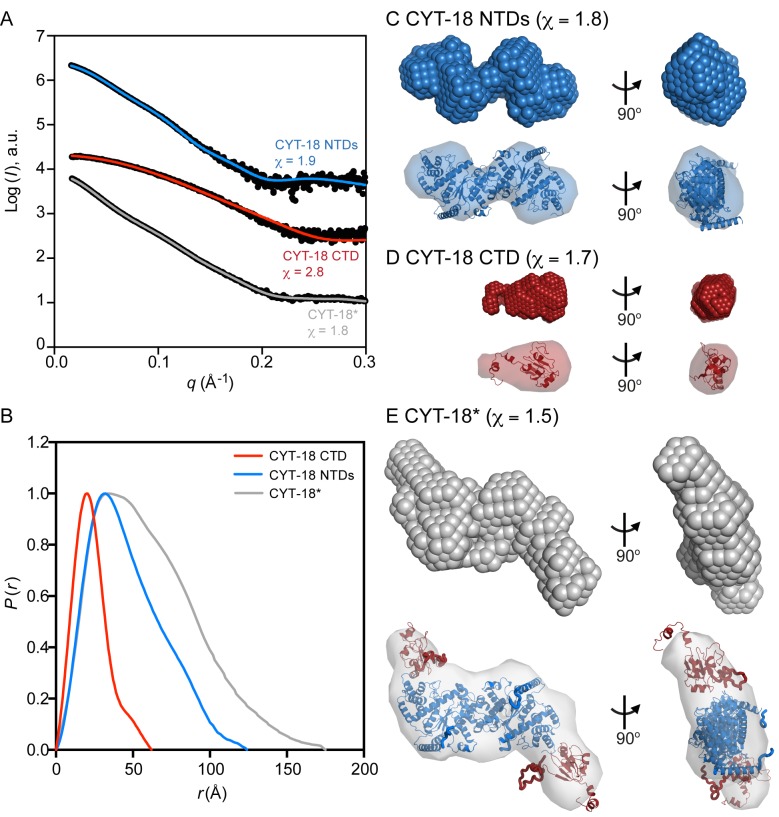
SAXS analysis of the CYT-18 protein constructs. (A) Scattering curves for the CYT-18 NTDs, CTD, and CYT-18*. The plots show the log of the scattering intensity (*I*, arbitrary units [a.u.]) as a function of momentum transfer (*q* = 4πsin(θ)/λ) and are displaced along the *y*-axis for visualization. The top and middle curves for CYT-18 NTDs and CTD show the SAXS data (black) overlaid with the expected scattering profiles calculated by CRYSOL from the CYT-18 NTDs crystal structure (blue, PDB:1Y42; χ = 1.9) or a CYT-18 CTD homology model based on the *A. nidulans* mtTyrRS CTD NMR structure (red, PDB:2KTL; χ = 2.8), respectively. The bottom curve for CYT-18* shows the SAXS data (black) overlaid with the calculated scattering profile for the CORAL model of this protein (gray; χ = 1.8). (B) Normalized pair distance distribution functions (*P(r)*) calculated from the scattering profiles by AUTOGNOM for CYT-18 CTD (red), CYT-18 NTDs (blue), and CYT-18* (gray). The *P(r)* curves for the CYT-18 NTDs and CTD are single peaks with a short tail, consistent with an elongated protein shape. (C–E) *Ab initio* models built by DAMMIN with the fit of the SAXS envelope to the corresponding high-resolution structure (CYT-18 NTDs), homology model (CYT-18 CTD), or CORAL model (CYT-18*) superimposed by SUPCOMB shown below the model [85]. The χ values shown in parentheses in the figure indicate the fit of the *ab initio* models to the scattering data.

Figure 2 shows SAXS curves for all three proteins, and Table 1 summarizes size parameters calculated from the SAXS curves, including the protein molecular weight; the maximum dimension of the particle (*D*_max_); and the radius of gyration (*R*_g_), which is the root mean square distance to the center of mass of a particle and provides an estimate of the overall particle size [42]. For all three proteins, Kratky plots of the SAXS data show a bell shape curve with a distinct peak, indicative of a folded globular protein (Figure S2).

**Table 1:** Size parameters determined from SAXS data.

Sample	Guinier *R*_g_ (Å)^a^	GNOM *R_g_* (Å)^a^	*D*_max_ (Å)^b^	MW – Sequence (kDa)^c^	MW – SAXS Standard (kDa)^d^	CRYSOL *R*_g_ (Å)	CRYSOL *D*_max_ (Å)
CYT-18 NTDs	35.6±0.2	36.7±0.1	123	89.6	84.4±4.7	35.2	125
CYT-18 CTD	17.7±0.4	18.1±0.1	62	14.8	13.3±0.7	17.0	55.9
CYT-18*	46.9±0.8	47.7±0.1	170	122	119±6	—	—
CYT18 NTDs+Twort RNA	39.2±0.1	39.3±0.1	137	161	—	39.1	134
CYT-18*+Twort RNA	41.9±0.1	41.8±0.1	146	194	—	—	—

a Radius of gyration calculated from the SAXS data by using the Guinier approximation or AUTOGNOM. Error estimates are the standard error of the linear regression and standard deviation, respectively.

b *D*_max_ values estimated by using the indirect transform program AUTOGNOM.

c Molecular weight calculated from protein and RNA sequence.

d Molecular weight calculated from SAXS data by comparison with the protein standard bovine serum albumin. Error estimates are for propagation of standard error calculated for *I*(0)/c from linear regression, where *I*(0) is the forward scattering intensity and c is the concentration of protein in g/l.

Focusing first on the CYT-18 NTDs protein, the scattering curve overlays well (χ = 1.9) with a theoretical scattering curve calculated from the previous CYT-18 NTDs crystal structure [33] by using the program CRYSOL (Figure 2A, top curve) [43]. The SAXS curve gave an estimated molecular weight of 84.4 kDa and *R*_g_ and *D*_max_ values of 35.6 and 123 Å, respectively, in good agreement with the molecular weight calculated from protein sequence (89.6 kDa) and with *R*_g_ and *D*_max_ values calculated from the crystal structure using CRYSOL (35.2 and 125 Å, respectively) (Table 1). The distance distribution function *P(r)* for the CYT-18 NTDs displays a single peak with a tail (Figure 2B), a pattern indicative of a protein having an elongated structure [44]. *Ab initio* models of the CYT-18 NTDs protein were built from the SAXS data by simulated annealing of either dummy atoms by DAMMIN or a chain-like ensemble of dummy residues by GASBOR (Figures 2C and S3, respectively) [45],[46]. The DAMMIN and GASBOR models show good fits to the experimental SAXS curve (χ = 1.8 for both models) and are similar in shape to each other and to the high-resolution structure as shown by the superposition of the crystal structure within the SAXS model envelopes. The final DAMMIN and GASBOR models are the result of analyzing multiple solutions and either averaging the models (DAMMIN) or picking the most representative one (GASBOR). The normalized spatial discrepancy (NSD) value, which describes the similarity between the different models produced by the programs, is low for both the DAMMIN and GASBOR models (0.63±0.03 and 1.10±0.02, respectively), indicating that the multiple solutions built by the programs are similar to each other (Table 2). Taken together, these results indicate that the conformation adopted by CYT-18 NTDs in solution is similar to that in the crystal structure [33].

**Table 2:** χ and NSD values for protein *ab initio* models.

Sample	DAMMIN		GASBOR	
	χ^a^	NSD^b^	χ^a^	NSD^b^
CYT-18 NTDs	1.8	0.63±0.03	1.8	1.10±0.02
CYT-18 CTD	1.7	0.49±0.02	2.2	0.78±0.02
CYT-18*	1.5	0.61±0.01	2.1	1.52±0.04

a The discrepancy value (χ) describes the fit of the *ab initio* model to the experimental scattering curve. χ values close to 1 indicate a good fit between the models and SAXS data.

b The NSD describes the similarity between different *ab initio* models produced by DAMMIN or GASBOR. A low value for the NSD (∼1) indicates the models are similar to each other.

The SAXS data for the isolated CTD overlays well with a theoretical scattering curve calculated from a homology model of CYT-18's CTD constructed from the NMR structure of the *A. nidulan* CTD using I-TASSER (χ = 2.8) (Figure 2A, middle curve) [47]. The molecular weight of 13.3 kDa estimated from the scattering data (Table 1) indicates that the CTD is monomeric in solution. The *R*_g_ and *D*_max_ values for the CTD calculated from the SAXS data (17.7 and 62 Å, respectively) are in good agreement with those for the I-TASSER model (17.0 and 55.9 Å, respectively) (Table 1). The DAMMIN and GASBOR models of the CYT-18 CTD (χ = 1.7 and 2.2, respectively) also superpose well with the homology model (Figures 2D and Figure S3, respectively). Thus, the SAXS analysis indicates that the CYT-18 CTD folds independently of the remainder of the protein and that the I-TASSER model provides a good representation of the structure of the CYT-18 CTD in solution. The latter finding validates the use of the I-TASSER model in building high-resolution structures of CYT-18* from the SAXS data (see below).

CYT-18* is the first CYT-18 protein to be investigated structurally that contains both the NTDs and CTD. The molecular weight for this protein estimated from the SAXS data is 119 kDa, confirming that CYT-18* is a dimer in solution (Table 1). The *R*_g_ and *D*_max_ values from the CYT-18* scattering data are 46.9 and 170 Å, respectively, both larger than that for the CYT-18 NTDs, as expected. *Ab initio* models of the CYT-18* homodimer indicate an open conformation with both CTDs extending outward from the NTDs (χ = 1.5 and 2.1 for the DAMMIN and GASBOR models, respectively) (Figures 2E and S3). A rigid-body model of CYT-18* was also built by CORAL, which constructs models that fit the SAXS data by combining high-resolution models of individual components, in this case the crystal structure of the CYT-18 NTDs and the I-TASSER model of the CTD (see above), with different conformations of flexible dummy residue linkers [48]. The CORAL model overlays well with the scattering curve (χ = 1.8) (Figure 2A, bottom curve) and superposes well into the SAXS envelopes of the *ab initio* models (Figures 2E and S3). These findings indicate that in the absence of intron RNA, CYT-18* adopts an S-shaped configuration with the two CTDs of the homodimer extending outward in opposite orientations.

### CYT-18 Forms a Compact Structure When Bound to a Group I Intron RNA

**Figure 3:**
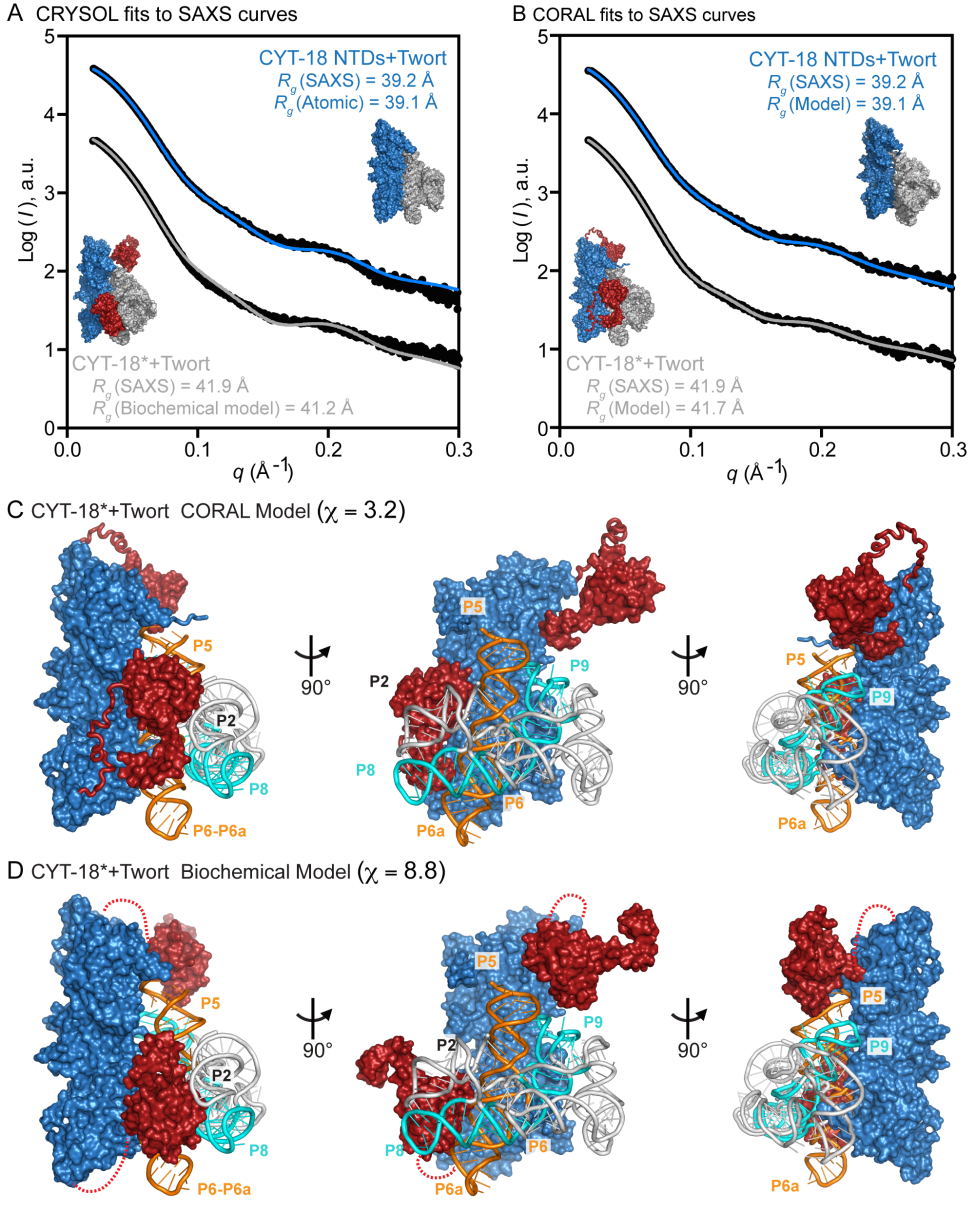
SAXS analysis and rigid-body modeling of CYT-18 proteins bound to the Twort ribozyme. (A, B) Scattering profiles of the CYT-18 NTDs and CYT-18* bound to the Twort ribozyme displaced along the logarithm axis for visualization. (A) shows CRYSOL fits of the SAXS curves (black) to theoretical scattering curves calculated from the CYT-18 NTDs+Twort co-crystal structure (blue; χ = 4.4) and CYT-18+Twort RNA model structure based on biochemical data [37] (gray; χ = 8.8). The CYT-18 NTDs+Twort RNA co-crystal structure and CYT-18+Twort RNA biochemical model structure are shown alongside the plots together with the *R*_g_ values calculated from the SAXS data and the structures. (B) shows fits of the SAXS curves (black) to the CORAL models of the CYT-18 NTDs+Twort (blue; χ = 2.2) and CYT-18*+Twort (gray; χ = 3.2). The CORAL models are shown alongside the plots together with the radii of gyration (*R*_g_) calculated from the SAXS data and the models. (C, D) CORAL and biochemical models of the CYT-18+Twort RNA complex, respectively. Three views of the models are shown with the CYT-18 NTDs colored blue and the two CTDs of the homodimer colored red. The unmodeled flexible linkers in the biochemical model are shown as dashed lines. The Twort group I intron RNA is depicted in gray cartoon representation with the P4–P6 domain in orange and the P3–P9 domain in cyan. Parts of intron RNA regions P2, P4–P5, P6–P6a, P8, and P9 are near and potentially interact with the CTDs.

The co-crystal structure of the CYT-18 NTDs bound to Twort intron RNA indicated that the RNA binds asymmetrically across the dimer interface and that the structure of the NTDs does not change substantially upon binding the intron RNA [22]. To elucidate interacting regions and conformational changes of the CTDs upon binding to the intron RNA, we obtained SAXS data for complexes of both the CYT-18 NTDs and CYT-18* bound to the same Twort group I intron RNA. The experimental scattering curve of the CYT-18 NTDs+Twort RNA complex overlays reasonably well with the scattering curve calculated from the co-crystal structure (χ = 4.4) (Figure 3A, top curve), and gave *R*_g_ and *D*_max_ values (39.2 and 137 Å, respectively) in agreement with those calculated from the co-crystal structure (39.1 and 134 Å, respectively) (Table 1). Likewise, a rigid-body model of the CYT-18 NTDs+Twort RNA complex built using CORAL shows a good fit to the experimental SAXS curve (χ = 2.2) (Figure 3B; top curve) and is similar in shape to the co-crystal structure (Figures 3A and 3B, compare insets above the top curves). These findings indicate that the CYT-18 NTDs+Twort RNA co-crystal structure is similar to the structure of the complex in solution and can be used as a component for structural modeling of the CYT-18*+Twort RNA complex from the SAXS data.

Finally, the scattering data for the Twort RNA complex with CYT-18*, which contains both the NTDs and CTD, gave *R*_g_ and *D*_max_ values of 41.9 Å and 146 Å, respectively (Figure 3A, bottom curve; Table 1). The relatively small difference between these values and those for the CYT-18 NTDs+Twort complex (*R*_g_ and *D*_max_ values of 39.2 Å and 137 Å, respectively; Table 1) suggests that CYT-18* forms a compact complex with the RNA in which the CTDs make a smaller than expected contribution to the overall particle dimensions. This conclusion was supported by ensemble optimization analysis using the program EOM, which generates a large random pool of conformations and picks an optimized ensemble that best fits the scattering data (see Materials and Methods). This optimized ensemble pool displayed a smaller, tighter range of *R*_g_ and *D*_max_ values than a random pool of protein-RNA conformations consistent with a compact rigid complex (Figure S4).

A rigid-body model of the CYT-18*+Twort RNA complex built using CORAL indicates that both CTDs are positioned near the Twort intron (Figure 3C). This CORAL model can be compared to a previous model of CYT-18*+Twort built using biochemical data (Figure 3D) [37]. While both the CORAL and biochemical models show that both CTDs are located near the intron RNA, the CORAL model better fits to the scattering data than does the biochemical model (χ = 3.2 and 8.8, respectively). In both models, the CTD of one subunit of the CYT-18 homodimer is close to and may interact with P2, P6–P6a, and P8 of the intron RNA, while the CTD of the other subunit is close to and may interact with P4–P5, and P9 of the intron RNA (Figure 3C and 3D). Considered together, the SAXS analyses indicate that upon binding a group I intron RNA, CYT-18* forms a compact complex in which both CTDs of the CYT-18 homodimer clamp down to interact with opposite ends of the group I intron RNA.

### The CYT-18 CTD Is a Non-specific RNA Binding Domain

**Figure 4:**
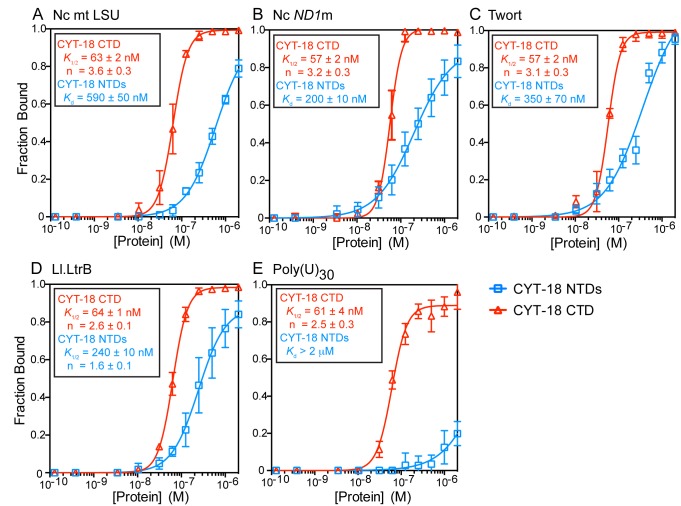
Equilibrium binding of CYT-18 NTDs and the CTD to various RNAs. Binding assays of CYT-18 NTDs (blue) or CTD (red) to the (A–C) *N. crassa* mt LSU (Nc mt LSU), *N. crassa ND1*m (Nc *ND1*m), and Twort ribozyme group I intron RNAs, respectively; (D) *L. lactis* Ll.LtrB group II intron RNA; and (E) poly(U)_30_. For the binding assays, ^32^P-labeled RNAs were incubated with increasing concentrations of protein at 25°C for 30 min, a time verified to be sufficient to reach equilibrium (see Materials and Methods). The plots show the fraction of RNA bound to a nitrocellulose filter as a function of protein concentration with the CYT-18 NTDs-group I intron binding data fit to hyperbolic curves and the CYT-18 NTDs-Ll.LtrB and all CYT-18 CTD-RNA binding data fit to sigmoidal curves. Dissociation constant (*K*_d_) or *K*_1/2_ values and Hill coefficient (n) are shown in (A–E) and are the mean for three experiments with the error bars indicating the standard deviation. Equilibrium-binding assays for the same proteins and RNAs at 37°C are shown in Figure S4.

To investigate how the RNA-binding properties of CYT-18's CTD enable it to interact with the two structurally distinct ends of a group I intron RNA, we analyzed the interaction of the CYT-18 NTDs and the isolated CTD with various RNAs by equilibrium-binding assays at 25°C and 37°C (Figures 4 and S5, respectively). The RNAs compared were three group I introns (the *N. crassa* mt large ribosomal subunit-ΔORF intron (Nc mt LSU); the *N. crassa* NADH dehydrogenase subunit 1-ΔORF intron (Nc *ND1*m); and the Twort intron RNA); a group II intron RNA (*Lactococcus lactis* Ll.LtrB-ΔORF; Ll.LtrB); and poly(U)_30_, which presumably lacks higher-order structure.

The binding curves for the CYT-18 NTDs to the Nc mt LSU, Nc *ND1*m, and Twort group I intron RNAs were best fit by hyperbolic functions with *K*_d_s ranging from 200 to 590 nM at 25°C (Figure 4A–4C) and 440 to 740 nM at 37°C (Figure S5A–S5C). The *K*_d_ values for the Nc *ND1*m intron are substantially higher than that calculated from previous *k*_off_ measurements, which assumed that the *k*_on_ of the construct lacking the CTD is the same as that for the wild-type protein (71±24 pM) [30]. This difference suggests that the CTD might make a major contribution to *k*_on_ by mediating the initial interaction with intron RNA substrates. At both temperatures, the strongest binding group I intron RNA was the Nc *ND1*m intron and the weakest was the Nc mt LSU intron, consistent with previous findings that the CTD is required for tight binding and splicing of the Nc mt LSU, but not the Nc *ND*1m intron [30]. At 25°C, the CYT-18 NTDs bound the Ll.LtrB group II intron RNA with a *K*_1/2_ = 240 nM, within the range of *K*_d_s for group I intron RNAs, but the binding curve was sigmoidal, with *n* = 1.6, suggesting cooperative and possibly non-specific binding (Figure 4D), whereas at 37°C, binding of the group II intron RNA was weaker (*K*_d_ = 440 nM) and the binding curve was hyperbolic (Figure S5D). The CYT-18 NTDs did not bind appreciably to poly(U)_30_ at either 25°C or 37°C (Figures 4E and S5E).

Surprisingly, the isolated CYT-18 CTD bound group I and II intron RNAs and poly(U)_30_ more strongly than did the CYT-18 NTDs and with similar affinities for all five RNAs tested (Figure 4). Indeed, the binding curves for the isolated CTD to these radically different RNAs were remarkably similar to each other, each being sigmoidal with *K*_1/2_s = 57–64 nM at 25°C and 51–77 nM at 37°C. These sigmoidal binding curves (*i.e.*, Hill coefficients (*n*)>1) suggest cooperative binding of two or more CTDs to each RNA. The ability of the isolated CTD to bind similarly to group I and II intron RNAs, as well as unstructured poly(U)_30_ indicates that it is a non-specific RNA binding domain.

### The Non-splicing *S. cerevisiae* mtTyrRS Binds Group I Introns Non-specifically Via Its CTD

The finding that the CYT-18 CTD is a non-specific RNA-binding domain led us to wonder whether an ancestral Pezizomycotina mtTyrRS might have initially bound group I intron RNAs non-specifically. To address this question, we turned to the *S. cerevisiae* (Sc) mtTyrRS, which is closely related to CYT-18 but branched from the Pezizomycotina mtTyrRSs prior to the evolution of splicing activity [23]. We compared two constructs that were expressed in *E. coli*: recombinant wild-type Sc mtTyrRS and a derivative lacking the CTD (Sc NTDs). We confirmed that both proteins are fully active in tyrosyl-adenylation, indicating correct folding of the catalytic domain (Figure S1A). Aminoacylation assays showed that the Sc mtTyrRS has higher activity with *E. coli* tRNA^Tyr^ than does CYT-18 (Figure S1B), possibly reflecting that the *E. coli* tRNA^Tyr^ and the Sc mt tRNA^Tyr^ are more similar to each other than to the Nc mt tRNA^Tyr^. All three tyrosyl-tRNAs share the same N73 identity element (A73) and anticodon, but differ in the N1-N72 identity element at the end of the acceptor stem (G-C in *E. coli* tRNA^Tyr^ and Sc mt tRNA^Tyr^, but A-U in Nc mt tRNA^Tyr^) and the length of the variable arm (13–14 nt in *E. coli* tRNA^Tyr^ and Sc mt tRNA^Tyr^, but unusually long at 16 nt in Nc mt tRNA^Tyr^) (Figure S6) [49]–[51].

**Figure 5:**
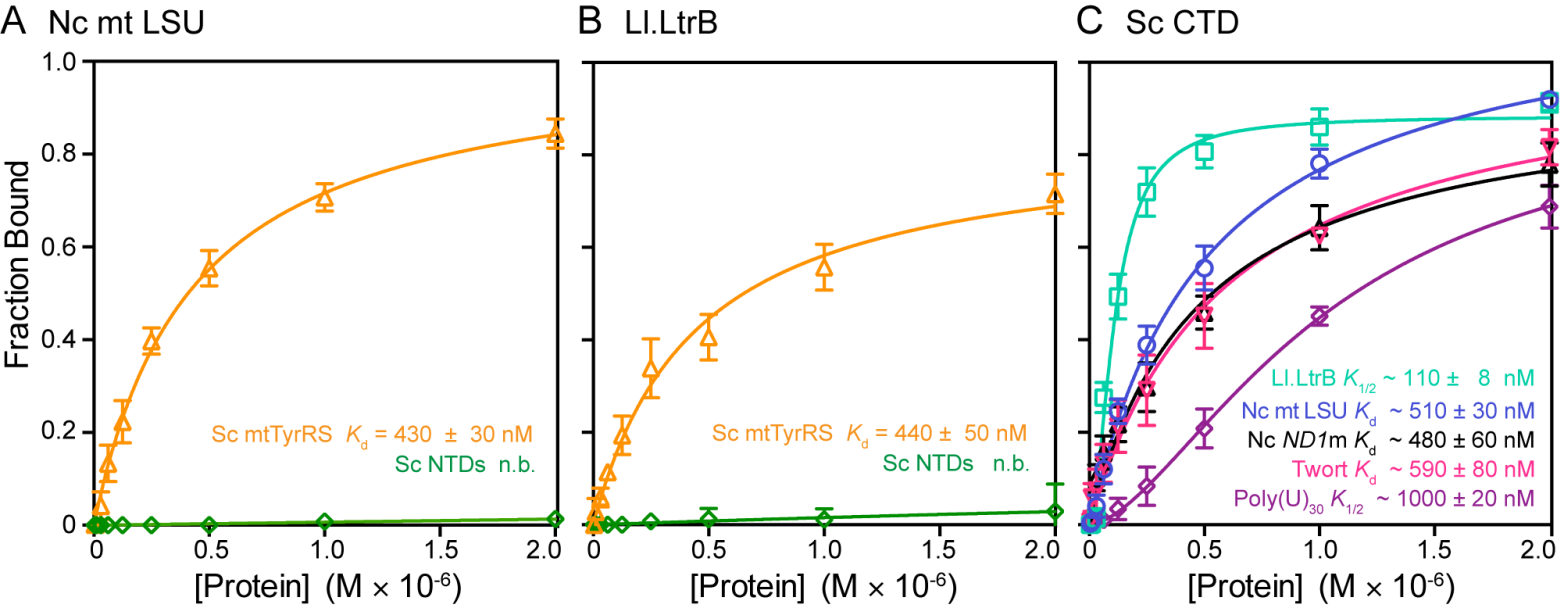
Equilibrium binding of the non-splicing *S. cerevisiae* mtTyrRS and its CTD to various RNAs. (A, B) Binding of Sc mtTyrRS (orange) and Sc NTDs (green) to the Nc mt LSU group I intron and *L. lactis* Ll.LtrB group II intron, respectively. (C) Binding of the CTD of the yeast mtTyrRS to the Nc mt LSU (blue), Nc *ND1*m (black), and Twort group I introns (pink); the Ll.LtrB group II intron (green); and poly(U)_30_ (purple). Binding assays were done at 25°C, as described in Materials and Methods. *K*_d_ or *K*_1/2_ values and Hill coefficients (n) are shown in boxes in (A–C) and are the mean for three experiments, with the error bars indicating the standard deviations. The Sc NTDs protein showed no detectable binding (n.b.) over the protein concentration range tested.

Equilibrium binding assays showed that the Sc mtTyrRS, although incapable of splicing group I intron RNAs [23], can bind both the Nc mt LSU group I intron RNA and Ll.LtrB group II intron RNA with *K*_d_s = 430 and 440 nM, respectively (Figure 5A and 5B), within the range of *K*_d_s for specific binding of CYT-18 NTDs to group I intron RNAs (see above). However, the similar affinity of the Sc mtTyrRS for the group I and group II intron RNAs suggests that this binding is non-specific. Strikingly, the ability of the Sc mtTyrRS to bind group I and II intron RNAs was entirely dependent upon its CTD, with the Sc NTDs protein showing no detectable binding of either intron RNA over the concentration range tested (Figure 5A and 5B).

To investigate if the Sc mtTyrRS CTD is a non-specific RNA-binding domain like CYT-18's CTD, we expressed and purified this domain separately including a small segment of the upstream linker region (denoted Sc CTD). We then assayed equilibrium binding of the Sc CTD to three group I introns (Nc mt LSU, Nc *ND1*m and Twort), a group II intron (Ll.LtrB), and poly(U)_30_ at 25°C. These assays showed that the Sc CTD is capable of binding all the RNAs tested with *K*_d_ or *K*_1/2_ values ranging from 110 nM to 1 µM (Figure 5C). The Sc CTD had the highest affinity for the Ll.LtrB group II intron RNA (*K*_1/2_ = 110 nM), which it bound in a cooperative manner (*n* = 1.8), and the lowest affinity for poly(U)_30_ (*K*_1/2_ = 1 µM; *n* = 1.6). Notably, for all RNAs tested, the non-specific binding by the isolated Sc mtTyrRS CTD is 1.5- to 15-fold weaker than the binding of CYT-18's CTD to the same RNA (*K*_1/2_s at 25°C = 57–64 nM) (Figure 4). Together, these findings indicate that the CTD of the Sc mtTyrRS is also a non-specific RNA-binding domain and that the Sc mtTyrRS binds both group I and group II intron RNAs non-specifically via its CTD.

### Splicing Properties of CYT-18/Sc mtTyrRS Chimeric Proteins

**Figure 6:**
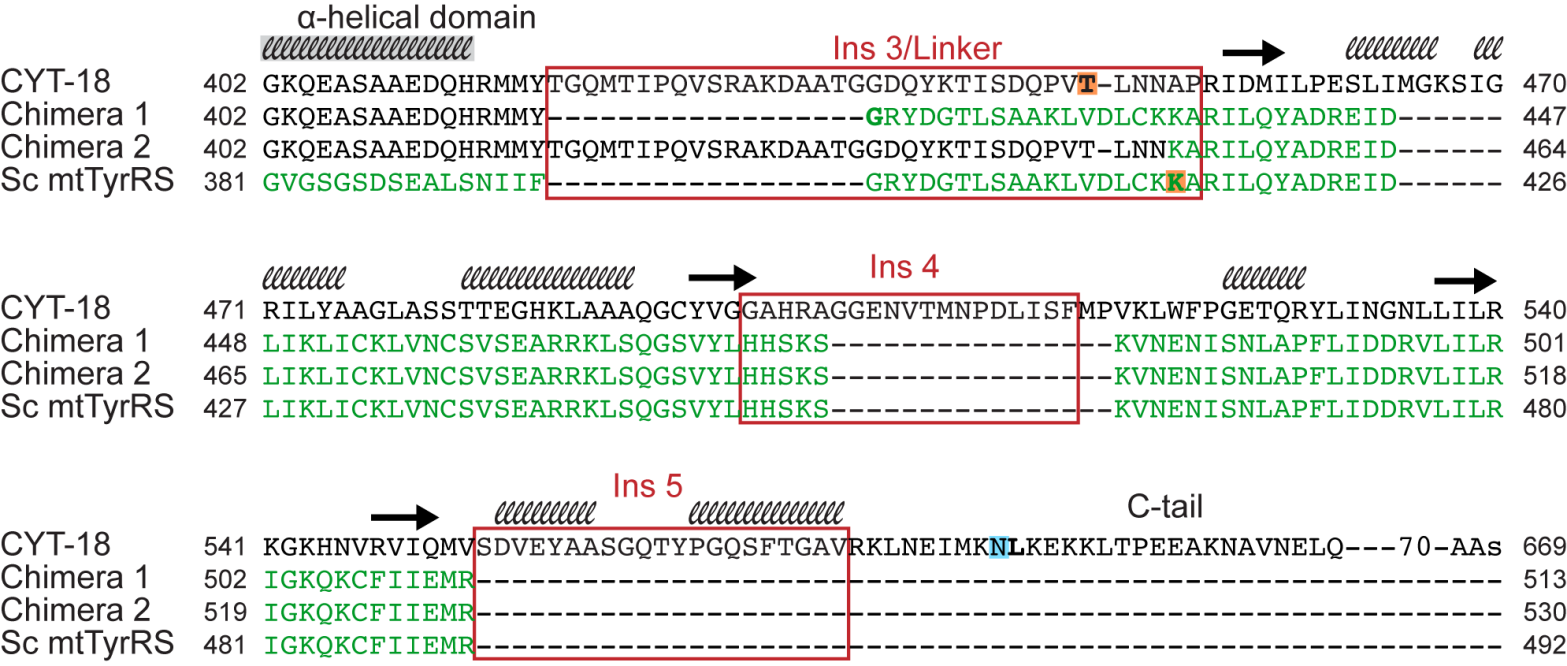
Sequence alignments showing the junction regions of CYT-18/Sc mtTyrRS chimeric proteins. CYT-18 and Sc mtTyrRS C-terminal regions starting from the end of the α-helical domain were aligned to the chimeric proteins using the multiple sequence alignment tool, MUSCLE [86]. The CYT-18 sequence is in black, and the Sc mtTyrRS sequence is in green, with regions corresponding to the CYT-18 CTD insertions in red boxes. The secondary structure of the CYT-18 CTD predicted by I-TASSER is shown above the CYT-18 CTD sequence as either black helices or arrows representing β-sheets. The first residues of the CYT-18 and Sc CTD constructs are highlighted in orange, and the last residue of the CYT-18* construct highlighted in blue.

**Figure 7:**
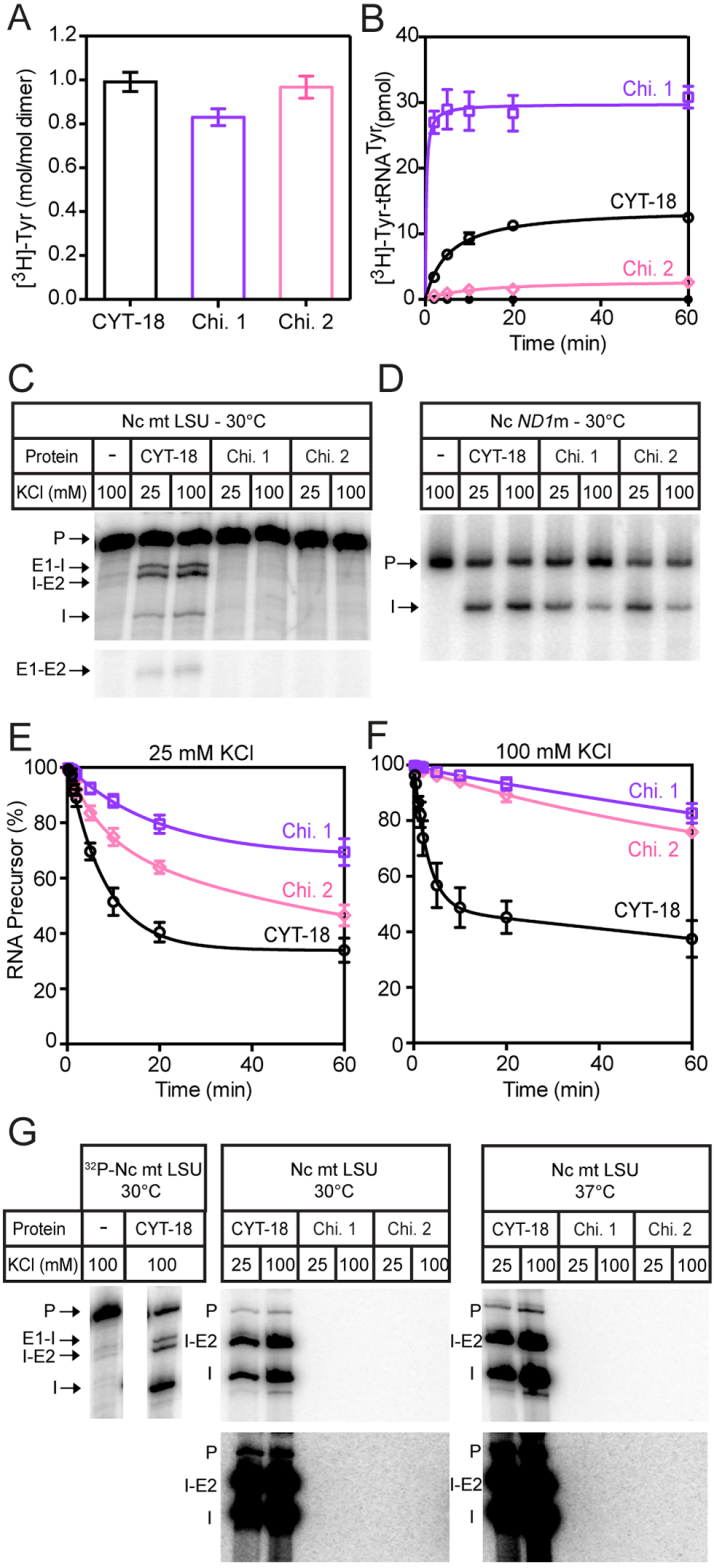
Biochemical analysis of CYT-18/Sc mtTyrRS chimeric proteins. Chimera 1 consists of the CYT-18 NTDs fused to the Sc mtTyrRS linker region and CTD, while chimera 2 consists of the CYT-18 NTDs and linker region (including Ins 3) fused to Sc CTD. Tyrosyl-adenylation, aminoacylation, and RNA-splicing assays were done at 30°C, as described in Materials and Methods. (A) Tyrosyl-adenylation activity is displayed as a bar graph showing the mean for three experiments, with the error bars indicating the standard deviation. (B) Aminoacylation assay showing the formation of [^3^H]-Tyr-tRNA^Tyr^ over a 60-min time course (black open circles, wild-type CYT-18; purple open squares, chimera 1; pink open diamonds, chimera 2; black closed circles, no protein). (C, D) End-point splicing assays of ^32^P-labeled precursor RNAs (200 nM) containing the Nc mt LSU and *ND1*m group I introns, respectively, with 100 nM protein and 1 mM GTP for 60 min at 30°C in splicing reaction medium containing 25 or 100 mM KCl. Additional end-point splicing assays with ^32^P-labeled precursor RNA at these protein and RNA concentrations at 25°C and 37°C are shown in Figure S7A and S7B. (E, F) Splicing time courses of ^32^P-labeled precursor RNA containing the *ND1*m intron (200 nM) with 100 nM protein and 1 nM GTP at 30°C in splicing reaction medium containing 25 mM or 100 mM KCl, respectively. The plots show disappearance of precursor RNA as a function of time (black open circles, wild-type CYT-18; purple open squares, chimera 1; pink open diamonds, chimera 2). (G) End-point splicing assays with unlabeled precursor RNA containing 200 nM Nc mt LSU intron and [α-^32^P]GTP (500 nM; 3,000 Ci mmol^−1^) with 500 nM protein. Reactions were incubated for 60 min at 30°C and 37°C. Darker exposures of the same gels are shown below. Minor labeled products in the CYT-18 lanes, including one co-migrating with precursor RNA and others migrating above precursor RNA (not shown), appear in time-course experiments after the 5′-labeled intron products and likely reflect secondary reactions catalyzed by group I intron RNAs (see [87]). The left panel shows splicing reactions for the same concentrations of wild-type CYT-18 protein and ^32^P-labeled precursor RNA at 30°C run in parallel as a control. Abbreviations: E1-E2, ligated exons; E1-I, 5′ exon+intron; I, excised intron; I-E2, intron+3′ exon; P, precursor RNA.

Finally, we investigated whether the Sc CTD could replace the CYT-18 CTD to promote group I intron splicing by making CYT-18/Sc mtTyrRS chimeric proteins. Two chimeric constructs were made differing in whether they contain the flexible linker region from CYT-18 or the Sc mtTyrRS (Figure 6). Chimera 1 consists of the CYT-18 catalytic and α-helical domains fused to the Sc CTD via the Sc mtTyrRS linker region, whereas chimera 2 contains the same CYT-18 NTDs fused to the Sc CTD via the CYT-18 linker, which includes Ins 3. Both chimeric proteins showed tyrosyl-adenylation activity similar to wild-type CYT-18, but displayed differences in aminoacylation activity (Figure 7A and 7B). Chimera 1, which contains both the Sc mtTyrRS linker and CTD, is similar to the yeast Sc mtTyrRS in having higher aminoacylation activity with *E. coli* tRNA^Tyr^ than does CYT-18, likely due to its better recognition of the bacterial tRNA (see above). By contrast, chimera 2, which contains the CYT-18 linker followed by the yeast mtTyrRS CTD, has substantially lower aminoacylation activity than CYT-18, indicating that the CYT-18 linker impairs charging of *E. coli* tRNA^Tyr^. The findings for chimera 1 indicate that higher TyrRS activity with *E. coli* tRNA^Tyr^ correlates with presence of the yeast CTD and linker, regions of the TyrRS that recognize the tRNA variable arm and anticodon stem, and not with the catalytic domain, which recognizes the acceptor stem [26],[28],[52]. The Sc mtTyrRS linker may contribute to the recognition of *E. coli* tRNA^Tyr^, either by contacting the tRNA directly or by facilitating binding of the CTD to the variable arm and/or anticodon stem.

To determine whether the Sc mtTyrRS CTD could function in splicing, we compared the ability of the chimeric proteins to splice the Nc mt LSU and *ND1*m group I introns, which do and do not require the CTD for splicing, respectively [30]. Group I introns splice via two sequential transesterification reactions initiated by the addition of guanosine nucleotide to the 5′ end of the intron, resulting in ligated exons and excised linear intron RNA with a non-coded G residue at its 5′ end [53]. The ability of the chimeric proteins to splice the Nc mt LSU and *ND1*m group I introns was assayed by using 200 nM ^32^P-labeled precursor RNA containing the introns, 100 nM protein, and unlabeled GTP at three different temperatures (25°C, 30°C, and 37°C) at either 100 mM KCl (the standard condition for CYT-18) or a lower salt concentration, 25 mM KCl (Figures 7C–7F, S7A, and S7B). The assays showed that the chimeric proteins could splice the Nc *ND1m* intron, which does not require the CTD, but could not splice the Nc mt LSU intron, which requires the CTD, under all conditions examined. The inability of the chimeric proteins to splice the Nc mt LSU intron was additionally confirmed by splicing assays done with higher protein concentration (500 nM; protein excess conditions) to compensate for potentially weaker binding of the intron RNA by the Sc CTD (Figure S7C), and by using a more sensitive assay in which [α-^32^P]GTP is incubated with unlabeled precursor RNA to label the 5′ end of the intron RNA during the first step of splicing (Figures 7G and S7D).

Notably, although the chimeric proteins were capable of splicing the *ND1*m intron (Figures 7D and S7B), they did so at a slower rate than full-length CYT-18, with the rate decreasing further at higher salt conditions (Figure 7E and 7F). The slower rate of *ND1*m intron splicing by the chimeric proteins is similar to that found previously for the CYT-18 NTDs alone [30], suggesting that it reflects a lack of contributing but nonessential interactions with the CTD. Interestingly, chimera 2 has higher splicing activity with the *ND1*m intron than does chimera 1, the reverse of what was found for TyrRS activity (Figure 7B). This finding suggests that the longer CYT-18-linker region, which is present in chimera 2 but not chimera 1, contributes to splicing activity. This contribution could involve either specific or non-specific interaction of Ins 3 with the group I intron RNA or increased conformational flexibility of the CTD due to expansion of the linker. Considered together, the findings for chimera 1 and chimera 2 indicate that although both the Sc mtTyrRS and CYT-18 CTDs bind group I intron RNAs non-specifically, the Sc CTD lacks further adaptations required for group I intron splicing activity.

## Discussion

Our results provide insight into the function of CYT-18's CTD and its contribution to the evolution of group I intron splicing activity, highlighting a role for non-specific binding interactions in the evolution of new RNA-binding functions. First, the SAXS analysis indicates that the CTDs of both subunits of the CYT-18 homodimer have a preferred orientation in solution extending outward in opposite directions from the NTDs, but move inward to bind opposite ends of a group I intron RNA. The CORAL model of CYT-18* bound to Twort intron RNA based on the SAXS data suggests that the CTD of one subunit binds the intron near P2, P6–P6a, and P8, while the CTD of the other subunit likely interacts with P4–P5 and P9 (Figure 3C). These interaction sites agree with a previous biochemical model based on directed hydroxyl-radical cleavage assays in which Fe-EPD with a cleavage radius of 25 Å was conjugated at two sites in the CTD (G493C and C494) [37]. These assays found cleavage sites in P6–P6a, P3–P8, and P5 in the Nc *ND1*m intron and P2, P4, and P6–P6a in the Nc mt LSU intron [37],[54]. The additional cleavages in the Nc mt LSU intron P2 helix, which is considerably longer than P2 of the Twort or Nc *ND1* introns, are consistent with its proximity to P8. The putative interaction sites between the CTDs and intron RNA in our CORAL model are also consistent with genetic experiments showing that CTD binding can suppress intron RNA mutations that impair long-range tertiary interactions P5-L9 and P2-L8 on opposite ends of the intron RNA [31].

The relatively fixed orientation of the CTDs in the free CYT-18 protein agrees with previous ^15^N-^1^H- two-dimensional NMR analysis showing that the CTDs of the full-length *A. nidulans* mt and *Geobacillus stearothermophilus* TyrRSs do not tumble independently in solution [37]. Nevertheless, the linker must be sufficiently flexible to allow the CTDs to bind to group I introns or tRNA^Tyr^ on opposite sides of the catalytic domain, and the SAXS analysis provides the first direct evidence for this conformational flexibility by showing the two CTDs of the homodimer swing downward from their starting position in the free protein to interact with different regions of a group I intron RNA.

We were surprised to find that the CTDs of both CYT-18 and the non-splicing yeast mtTyrRS are non-specific RNA-binding domains. The isolated CTDs of both proteins bind structured group I and group II intron RNAs, or the simple homopolymer, poly(U)_30_ with similar affinities, with this non-specific binding 1.5- to 15-fold stronger for the CYT-18 CTD than the yeast mtTyrRS CTD (see Results). The non-specific RNA-binding activity of the TyrRS CTDs may contribute to its function in aminoacylation by augmenting its specific-binding interactions with tRNA^Tyr^, which include recognition of the variable arm and anticodon bases [28],[55]. Likewise, the high non-specific binding activity of the CYT-18 CTD does not preclude and may bolster specific-binding interactions of this domain with group I intron RNAs. The latter could result either from further adaptive evolution of the CTD or simply from positioning of the CTD on the intron RNA via specific binding of the NTDs.

Although non-specific RNA binding was unexpected for an aaRS domain involved in tRNA recognition, yeast and higher eukaryotic aaRSs have been shown previously to have appended non-specific RNA-binding domains that are not present in their bacterial counterparts and contribute to aminoacylation efficiency. Thus, the yeast glutaminyl-tRNA synthetase (GlnRS) has an N-terminal non-specific RNA binding domain, which when fused to a bacterial GlnRS enabled it to functionally replace the yeast enzyme *in vivo*, as did fusion of the yeast Arc1 protein, a non-specific RNA-binding protein that ordinarily helps mediate tRNA/aaRS interactions in *trans* [56],[57]. Similarly, some higher eukaryotic aaRSs have tandem repeats of a small non-specific RNA-binding motif that enhances tRNA binding [58]. These non-specific RNA-binding domains are thought to act by adding sufficient binding energy to compensate for relatively weak specific binding interactions of aaRSs with tRNA substrates, similar to the augmentation of specific binding of tRNA and intron RNA substrates suggested above for the TyrRS CTD.

Notably, the ribosomal protein S4-like fold, which forms the core of bacterial and mitochondrial TyrRS CTDs, has been identified previously as an ancient RNA-binding domain. This domain is found in all three kingdoms of life in a variety of proteins that bind structurally different RNAs, including two families of pseudouridine synthetases, a family of predicted RNA methylases, an RNA-modification enzyme with both pseudouridine synthetase and cytidine deaminase activity, threonyl-tRNA synthetases, and a heat-shock protein [59]–[61]. The S4-like fold consists of two α-helices arranged as a helical hairpin packed against three or four β-sheets. Connecting two of the β-sheets is a characteristic L-shaped loop, which together with the two α-helices is termed the αL motif. This motif generally contains clusters of basic and polar residues that are capable of interacting with various nucleic acid substrates in the different S4-like fold containing proteins. In TyrRSs, the αL motif interacts in a region between the variable and anticodon arms [61],[62]. We suggest that the inherently high non-specific RNA-binding affinity of the S4-like fold was the key factor enabling it to evolve interactions with different RNA substrates in the course of evolution. Indeed, the fungal mtTyrRSs provide a dramatic example of a case in which the S4-like fold of a single enzyme may bolster specific-binding interactions with three different regions of two different RNA substrates, a mt tRNA^Tyr^ and a group I intron RNA.

Although we suggest that the non-specific binding of the CTD played a key role in initial interaction with group I intron RNAs, the CTDs of present-day fungal mtTyrRS appear to have evolved specific interactions with group I intron RNAs. Thus chimeric proteins containing the CYT-18 NTDs linked to the yeast CTD can efficiently aminoacylate *E. coli* tRNA^Tyr^, as well as splice the Nc *ND1* intron, which requires only the NTDs [30]. However, the chimeric proteins splice the Nc *ND1m* intron less efficiently than full-length CYT-18 at a rate expected for loss of contributing CTD interactions, and they are unable to splice the Nc mt LSU intron, which requires the CTD [30]. Additional adaptations of the CYT-18 CTD required to promote splicing may include RNA-binding contacts by Ins 3–5, which are found in the CTDs of splicing-competent Pezizomycotina mtTyrRS, but not in the Sc mtTyrRS [37]. Both the previous biochemical model of CYT-18*+Twort [37] and the new CORAL model based on the SAXS data (Figure 3C) place Ins 4 and 5 in position to bind group I intron RNAs.

Since the discovery of the splicing function of CYT-18 [17], there have been numerous additional examples of aaRSs that have acquired new functions unrelated to translation, in most cases via addition of non-catalytic domains [63],[64]. The acquisition of these new domains and functions is thought to reflect that aaRS are ancient essential enzymes whose presence early in evolution of the cell provided a robust scaffold for the addition of new structural elements [65]. In archael and eukaryotic TyrRSs, the N-terminal catalytic domain is followed by a different anticodon-binding domain, known as the C-W/Y domain, which is homologous to the anticodon-binding domain of TrpRSs [66]. Two additional structural elements were acquired during the evolution of higher eukaryotes and function in receptor-mediated signaling pathways associated with angiogenesis: the ELR motif in the catalytic domain and a C-terminal EMAP II-like domain, which has non-specific RNA-binding properties [63],[67]–[69]. The ELR motif is on the intron-binding side of the catalytic domain [70] and incorporated in the same α-helix as Ins1 in the fungal mtTyrRS, suggesting that this region may be a particularly robust location for insertion of new functional elements.

**Figure 8:**
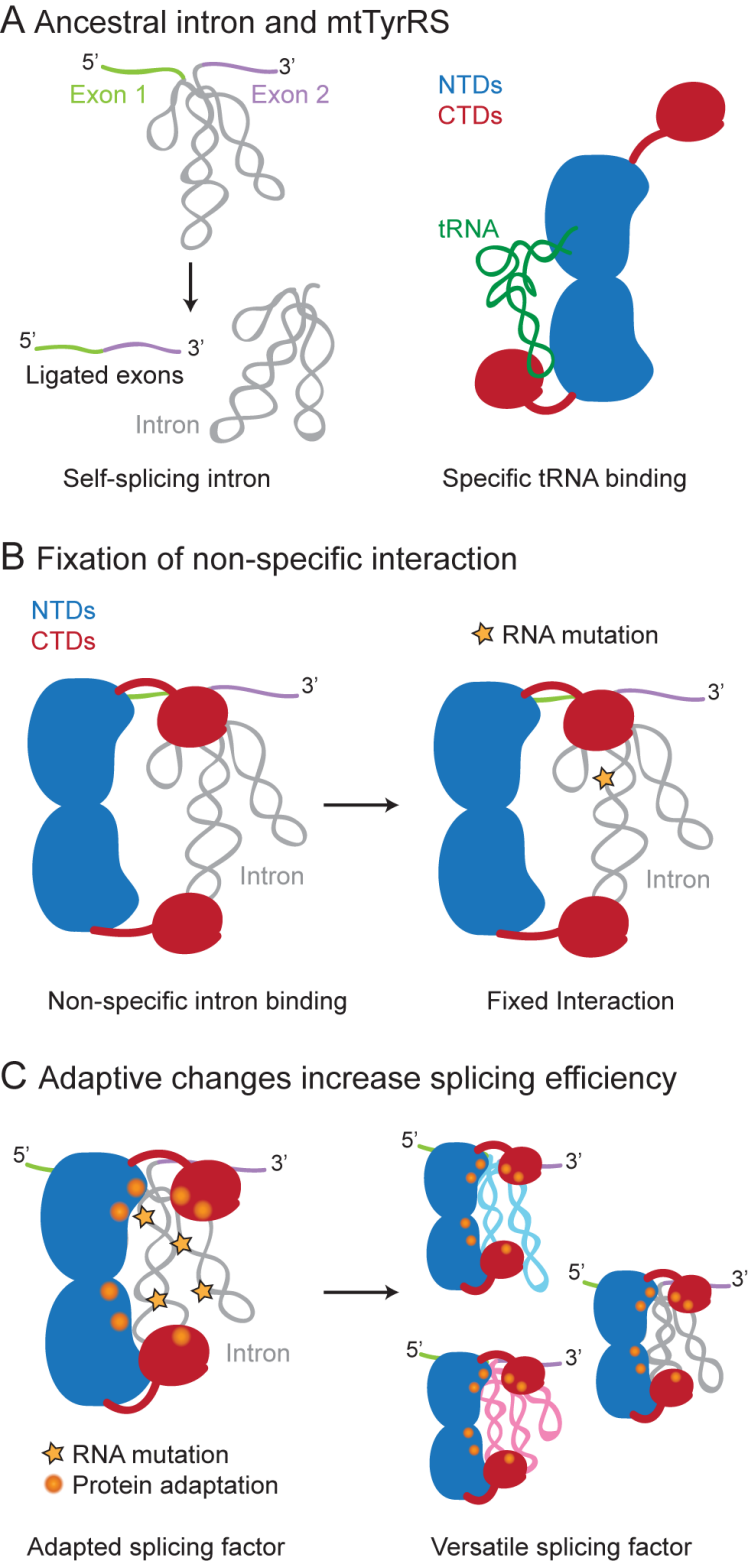
Model for the evolution of the RNA splicing activity of fungal mtTyrRSs via non-adaptive non-specific RNA-binding by the CTD. (A) A Pezizomycotina ancestor contained a self-splicing group I intron and an mtTyrRS, which functions in aminoacylation by specifically binding tRNA^Tyr^. (B) The ancestral mtTyrRS could bind non-specifically to the group I intron and other RNAs via its CTD, and its interaction with a group I intron RNA was fixed by an intron RNA mutation (orange star) that made it dependent upon the binding of the mtTyrRs for formation or stabilization of the active ribozyme structure. (C) Once fixed, this initial non-specific interaction was elaborated by further adaptive mutations in both the protein (orange spheres) and intron RNA (orange stars) that increased both the efficiency and protein-dependence of RNA splicing. The adaptive mutations in the fungal mtTyrRSs resulted in specific binding of the conserved tertiary structure of the group I intron RNA catalytic core, conferring the ability to bind and splice additional group I introns (blue, pink).

Finally, our results provide evidence that non-specific binding can play a key and perhaps widespread role in pre-adaptive interactions that lead to the evolution of new RNA-binding functions of proteins. For the group I intron splicing activity of fungal mtTyrRSs, our findings suggest a scenario outlined in Figure 8 in which an initial non-specific interaction between the CTD of an ancestral mtTyrRS and a group I intron RNA was fixed by an intron RNA mutation that made formation of active ribozyme structure dependent upon interaction with the protein. After the interaction was fixed, the mtTyrRS and group I intron were forced to co-evolve, with further adaptive mutations in the protein leading to specific binding of both the catalytic domain and CTD to the intron RNA. These specific-binding interactions extended the intron RNA-binding surface, both increasing the efficiency of splicing and permitting additional mutations in the intron RNA that made it more dependent upon the protein for structural stabilization. RNA-editing enzymes such as APOBEC1, which evolved from enzymes that acted on mononucleotide substrates, may be additional examples of constructive neutral evolution in which a relatively non-specific pre-adaptive interaction with an RNA substrate was fixed by a deleterious mutation, in this case one that could be corrected by RNA editing, and then elaborated by further adaptive mutations [71],[72]. Indeed, a similar evolutionary pathway may have been used more generally for other RNA-modification enzymes, including the ones mentioned above that contain an S4-like non-specific RNA-binding domain.

Beyond the initial pre-adaptive phase, the extensive structural data for the interaction of fungal mtTyrRSs with group I intron RNAs provide strong evidence for a ratchet-like process in which multiple adaptive mutations, including six different Peziomycotina-specific insertions, led to the evolution of an efficient splicing apparatus for group I introns. It is highly unlikely that the multiple adaptive mutations in the protein leading to an extensive group I intron-binding surface occurred in one step. The surprising finding that the structural adaptations of the mtTyrRS catalytic domain utilized a non-tRNA-binding surface could reflect that the tRNA-binding site in the catalytic domain could not be easily modified to function in group I intron splicing without inhibiting mtTyrRS activity, which is essential in an obligate aerobe. Additionally, the non-tRNA-binding side of the catalytic domain may have had a pre-existing auxiliary RNA-binding function, as found for some aaRSs [73],[74]. By contrast to the catalytic domain, the regions of the CTD needed for splicing activity overlap tRNA-binding regions requiring co-evolution with both the intron RNA and mt tRNA^Tyr^. Indeed, the unusually long variable arm of Pezizomycotina mt tRNA^Tyr^s (see Results) (Figure S6) may be an example of a feature that co-evolved with the CTD to allow it to better accommodate group I intron RNAs [37]. We also note that although the initial interaction of an ancestral fungal mtTyrRS likely involved a single group I intron RNA, perhaps the mt LSU intron, which is dependent upon the mtTyrRS for splicing in all Pezizomycotina fungi examined [23], the fungal mtTyrRSs ultimately evolved to function in splicing multiple group I introns by recognizing the conserved phosphodiester backbone structure of the catalytic core. This binding mode has the evolutionary advantages of enabling the fungal mtTyrRSs to coordinate the splicing of multiple group I introns as well as the ability to accommodate new group I introns that invade genomes as mobile genetic elements.

## Materials and Methods

### Recombinant Plasmids

Recombinant plasmids used for protein expression in *E. coli* are derivatives of the phage T7 promoter-driven expression vectors pET3a, pET11a, or pET11d (EMD Millipore). pEX560, which expresses a wild-type CYT-18 protein (amino acids 33–669), contains the *cyt-18* ORF (nucleotides 97–2,010) cloned downstream of the T7 promoter in pET3a [29]. pCYT18/ΔC-tail, which expresses CYT-18* (C-terminal truncation of the non-essential C-tail; amino acids 584–669), was derived from pEX560 by introducing three stop codons (TAATAGTAG) after Leu583 by site-directed mutagenesis (QuikChange; Agilent Technologies). pHISTEV602 expresses the CYT-18 NTDs (C-terminal truncation of both the CTD and C-tail; amino acids 424–669), with an N-terminal tobacco etch virus (TEV) protease-cleavable 6× His-tag. It was constructed by PCR of pEX560 using primers that amplify nucleotides 97–1,251 of the CYT-18 ORF and append NcoI and BamHI sites, and then cloning the resulting PCR product between the NcoI and BamHI sites of pET11d. pCYT18-CTD, which expresses the CYT-18 CTD (amino acids 448–583) with an N-terminal TEV-cleavable 6× HIS-tag, was constructed by PCR of pEX560 using primers that amplify nucleotides 1,342–1,749 of the CYT-18 ORF and append NdeI and BamHI sites, and then cloning the resulting PCR product between the NdeI and BamHI sites of pET11a. All CYT-18 expression constructs lack the mt targeting sequence (amino acids 1–32). Wild-type CYT-18 and CYT-18* have an extra N-terminal methionine, while CYT-18 NTDs and CTD have an extra N-terminal glycine resulting from TEV-protease cleavage of the N-terminal 6× His-tag.

pHISTEVScTyrRS, which expresses the full-length mature *S. cerevisiae* mtTyrRS with an N-terminal TEV-cleavable 6× HIS-tag, contains Sc mtTyrRS codons 38–492 (lacking the mt target sequence; amino acids 1–37) cloned between the Nco1 and BamHI sites of pET11d [23]. pHISTEVSc/ΔCTD expresses Sc mtTyrRS lacking the CTD (denoted Sc NTDs) and was derived from pHISTEVScTyrRS by using site-directed mutagenesis to add three stop codons (TAATAATAA) after Asp400. pMAL-ScCTD, which expresses the Sc mtTyrRS CTD (denoted Sc CTD), contains Sc CTD codons 414–492 cloned between the BamHI and HindIII sites of pMAL-c2t [75], a derivative of plasmid pMAL-c2x (New England Biolabs) that expresses the protein with an N-terminal maltose-binding protein tag followed by a TEV-protease site.

Chimeric proteins containing the N-terminal catalytic domain of CYT-18 and the CTD of the Sc mtTyrRS were made by overlap PCR. Chimera 1 contains the CYT-18 NTDs (amino acids 33–417) fused to the Sc mtTyrRS flexible linker and CTD (amino acids 397–492). Chimera 2 contains the CYT-18 NTDs and linker including Ins 3 (amino acids 33–451) fused to the Sc mtTyrRS CTD (amino acids 416–492). The chimeric protein ORFs were cloned between the BamHI and HindIII sites of pMAL-c2t (see above), enabling the expression of fusion proteins with an N-terminal TEV-protease cleavable maltose-binding protein tag.

Recombinant plasmids used for *in vitro* transcription contain group I or II introns cloned downstream of a phage T3 or T7 promoter. pBD5a contains the *N. crassa* mt large subunit rRNA-ΔORF (Nc mt LSU) intron cloned downstream of a T3 promoter in pBS(+) [19]. Transcription of pBD5a linearized with BanI yields a 503-nt RNA containing a 65-nt 5′ exon, the 388-nt mt LSU intron, and a 50-nt 3′-exon. pND1m contains the *N. crassa* NADH dehydrogenase subunit 1-ΔORF (Nc *ND1*m) intron cloned downstream of a T7 promoter in pUC18 [18]. Transcription of pND1m linearized with NdeI yields a 209-nt RNA containing a 6-nt 5′ exon, the 196-nt *ND1* intron, and a 7-nt 3′ exon. pTWORT-P2 contains a ribozyme derivative of a group I intron of the *Staphylococcus aureus* bacteriophage Twort *orf*142 gene (intron nucleotides 9-250) cloned downstream of a T7 promoter in pUC19 [76]. Transcription of pTWORT-P2 linearized with EarI yields a 242-nt transcript of the Twort ribozyme. pSSltrBΔA contains a derivative of the *L. lactis* Ll.LtrB-ΔORF intron with a deletion of the branch-point nucleotide to prevent splicing during binding assays cloned downstream of a T7 promoter in pUC19 [77]. Transcription of a DNA template made by PCR of the pSSltrBΔA plasmid (forward primer 5′-ATGAATTCTAATACGACTCACTATAGGGTTATAATTATCCTTACACATCCATAAC and reverse primer 5′-CGCTGCAGAATTGATATCAAAAATGATATG) yields an 807-nt RNA containing a 28-nt 5′ exon, the 749-nt intron, and a 30-nt 3′ exon.

### Protein Expression and Purification

Proteins were expressed from the recombinant plasmids indicated above in *E. coli* HMS174(DE3) (CYT-18, CYT-18*, and CYT-18 NTDs); BL21(DE3) (CYT-18 CTD, chimera 1, and chimera 2); or Rosetta 2(DE3) (EMD Millipore) (Sc mtTyrRS, Sc NTDs, and Sc CTD). Overnight cultures of fresh transformants were inoculated into LB media, and the proteins expressed via auto-induction [78]. Cells expressing CYT-18 and CYT-18* were grown at 35°C overnight with shaking at 260 rpm. Cells expressing all other proteins were grown at 37°C for 4 h then shifted to 25°C overnight with shaking at 260 rpm.

Wild-type CYT-18 and CYT-18* were purified as described [22],[27]. Briefly, cells were lysed by incubation with lysozyme at 1 mg/ml for 30 min followed by polyethyleneimine precipitation to remove nucleic acids, and ammonium sulfate precipitation [27]. The ammonium sulfate pellet was dissolved in 500 mM KCl, 25 mM Tris-HCl (pH 7.5) and then dialyzed overnight in 25 mM KCl, 25 mM Tris-HCl (pH 7.5). The protein was purified from the dialysate by using a HiTrap SP XL cation exchange column (GE Healthcare Life Sciences), followed by a size-exclusion column (HiLoad 16/60 Superdex 200; GE Healthcare Life Sciences) [22].

The 6× HIS-tagged proteins CYT-18 NTDs, CYT-18 CTD, Sc mtTyrRS, and Sc NTDs were purified similarly, except that the ammonium sulfate pellet was dissolved in 500 mM KCl, 25 mM Tris-HCl (pH 7.5), and 30 mM imidazole, and the proteins were purified by nickel-affinity chromatography using a HisTrap HP column (GE Healthcare Life Sciences) [23], followed by TEV protease-cleavage of the 6× HIS-tag in dialysis buffer (500 mM KCl, 25 mM Tris-HCl [pH 7.5], 5 mM DTT) to remove imidazole. The proteins were then further purified by an additional round of nickel-affinity chromatography, followed by size-exclusion chromatography (HiLoad 16/60 Superdex 200; GE Healthcare Life Sciences).

The maltose-binding protein (MalE) fusions MalE-ScCTD, MalE-chimera 1, and MalE-chimera 2 were purified by polyethyleneimine precipitation of nucleic acids, as described above for CYT-18, and then loaded onto an amylose affinity column (New England Biolabs) in buffer containing 25 mM Tris-HCl (pH 7.5), 500 mM KCl, 1 mM DTT, 1 mM EDTA, and 10% glycerol followed by elution with 10 mM maltose in the same buffer. The proteins were further purified using a heparin-sepharose column (HiTrap heparin HP column; GE Healthcare Life Sciences) in 300 mM KCl, 25 mM Tris-HCl (pH 7.5), 1 mM DTT, and 1 mM EDTA and eluted with a salt gradient of 300 mM to 1.5 M KCl in the same buffer. The final purification step was size-exclusion chromatography (HiLoad 16/60 Superdex 200; GE Healthcare Life Sciences) in 25 mM Tris-HCl (pH 7.5), 200 mM KCl, and 10% glycerol.

Proteins used for SAXS were stored in buffer containing 100 mM KCl, 5 mM MgCl_2_, 10 mM Tris-HCl (pH 7.5), 5% glycerol at −80°C. Proteins used for biochemical assays were dialyzed into 100 mM KCl, 25 mM Tris-HCl (pH 7.5), and 50% glycerol and stored at −80°C. Protein yields ranged from 14 to 44 mg/l (monomer concentrations), and all proteins were >99% pure as judged by SDS-polyacrylamide gels stained with Coomassie blue. Protein concentrations were determined by measuring A_280_ under denaturing conditions (6 M guanidine hydrochloride). Concentrations of wild-type CYT-18, the Sc mtTyrRS, and C-terminal truncations of these proteins refer to the homodimer, while CTD concentrations refer to the monomer.

### Preparation of RNA Substrates for SAXS and Biochemical Assays

Intron-containing RNA substrates for SAXS and biochemical assays were transcribed from the linearized recombinant plasmids indicated above. The Twort intron for SAXS was synthesized by large-scale *in vitro* transcription reactions (10–30 ml) with T7 polymerase at 37°C in reaction buffer containing 40 mM Tris-HCl (pH 8.1), 1 mM spermidine, 10 mM DTT, 8 mM NTPs, and 15 mM MgCl_2_. Transcription reactions were incubated at 37°C for 8 h and terminated by adding 50 mM EDTA followed by extraction with phenol-chloroform-isoamyl alcohol (25∶24∶1; phenol-CIA). The RNA was then purified through a 5-ml HiTrap desalting column (GE Healthcare Life Sciences) and a size exclusion column (HiLoad 16/60 Superdex 200; GE Healthcare Life Sciences). T7 RNA polymerase for the large-scale transcriptions was expressed with an N-terminal 6× HIS-tag from pRC9 and purified as described [79].

^32^P-labeled Nc mt LSU, Nc *ND1*m, and Twort RNAs for equilibrium-binding assays were synthesized by using a MAXIscript transcription kit (Life Technologies), with the concentration of unlabeled UTP changed from that recommended in the manufacturer's protocol (0.5 mM) to 10 µM UTP to obtain higher specific activity transcripts (300 Ci/mmol). The Ll.LtrB group II intron was synthesized by using a mutant T7 polymerase that can read through a T7 polymerase transcription termination site within the intron [80] in reaction medium containing 40 mM Tris-HCl (pH 7.9), 6 mM MgCl_2_, 10 mM DTT, 2 mM spermidine, 1 mM GTP, 1 mM CTP, 1 mM ATP, 250 nM UTP, and 1 µM [α-^32^P]UTP (3,000 Ci mmol^−1^; Perkin Elmer). After transcription and DNase treatments (MAXIscript transcription kit; Life Technologies), transcripts were purified by extraction with phenol-CIA, followed by gel filtration through two consecutive 1-ml Sephadex G-50 columns (Sigma-Aldrich).

Intron-containing RNA substrates for splicing reactions were transcribed from linearized DNA template using a MEGAscript transcription kit (Life Technologies) with 1 µCi [α-^32^P]UTP (3,000 Ci mmol^−1^; PerkinElmer) added for standard ^32^P-labeled substrates and 3 µCi [α-^32^P]UTP (3,000 Ci mmol^−1^) added for higher specific activity subtrates (Figures 7C, S7A, and S7C). The Nc mt LSU intron substrate was synthesized by *in vitro* transcription of pBD5a (BanI digested) using a MEGAscript T3 kit, while the Nc *ND1*m intron substrate was synthesized by *in vitro* transcription of pND1m (NdeI digested) using a MEGAscript T7 kit. The intron RNAs were purified as described above.

The poly(U)_30_ oligonucleotide used for binding assays was synthesized and HPLC-purified by Integrated DNA Technologies. The oligonucleotide was dissolved in 10 mM HEPES (pH 7.5), 1 mM EDTA and stored at a concentration of 25 µM. For equilibrium-binding assays, 25 pmoles of the oligonucleotide was 5′-end labeled with [γ-^32^P]ATP (3,000 Ci mmol^−1^; PerkinElmer) using T4 kinase (New England Biolabs) and then purified by phenol-CIA extraction followed by desalting through a Sephadex G-25 column.

### SAXS Data Collection and Analysis

Proteins and RNAs used for SAXS analysis were prepared as described above. RNA-protein complexes were formed by mixing protein dimer and RNA at a 1∶1 molar ratio in 1.2 ml of 100 mM KCl, 5 mM MgCl_2_, 10 mM Tris-HCl (pH 7.5), and 5% glycerol. After incubation at room temperature for 15 min, RNP complexes were purified by size-exclusion chromatography (Hi-Load 16/60 Superdex 200 column; GE Healthcare Life Sciences) in the same buffer. RNP complexes and proteins for SAXS were concentrated by using Amicon Ultra-4 centrifugal filter units (EMD Millipore) and frozen for storage at −80°C. The size-exclusion chromatography column buffer was used as a solvent blank for SAXS.

SAXS data were collected on beamline 12-ID-C at the Advanced Photon Source (Argonne, Illinois). Each sample had 20 1-s exposures taken at a sample-to-detector distance of 2.0 m, covering a momentum transfer range of 0.007<*q*<0.35 Å^−1^. Samples were continuously passed through the beam using a flow-cell to minimize radiation damage. The 20 consecutive exposures were compared and showed no change in scattering intensity, indicating no radiation damage. Radially averaged scattering data were buffer subtracted and analyzed by using ATSAS [42] and IGOR-Pro (WaveMetrics). Scattering curves were displayed as the scattering intensity (*I(q)*) as a function of momentum transfer *q* = (4πsinθ)/λ, where λ is the wavelength of the incident X-ray beam and θ is half the angle between the incident and scattering radiation. SAXS data were obtained for least three different concentrations of each protein and checked for aggregation and interparticle interference by examination of the Guinier region [81]. Guinier plots (log(*I*(*q*)) versus log(*q*)) were checked for linearity in the Guinier region, a diagnostic of sample quality. For globular proteins, the Guinier approximation is valid for *qR_g_*<1.3. The *q* range used for SAXS analysis was 0.015<*q*<0.3 Å^−1^ for CYT-18 protein constructs and 0.02<*q*<0.3 Å^−1^ for CYT-18+Twort complexes. The *I*(0) (extrapolated forward scattering at zero angle) and *R*_g_ (radius of gyration) were evaluated using the Guinier approximation for scattering intensity (*I*(*q*)) according to the equation:
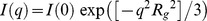

*I*(0) and *R*_g_ were also computed from the scattering curve by using the indirect Fourier transform program AUTOGNOM, which additionally provides an estimate of the maximum particle dimension (*D*_max_) from the distance distribution function *P(r)* [42]. The *R*_g_ values determined by using the Guinier approximation were consistent with those determined by AUTOGNOM. Molecular weights were calculated by comparing the extrapolated forward scattering at zero angle, *I*(*0*), with that of a protein standard, bovine serum albumin (BSA), by using the equation:
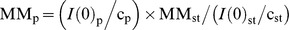
where MM_p_ and MM_st_ are the molecular weights of the protein sample and protein standard, respectively, c_p_ and c_st_ are their concentrations in g/l, and *I*(0)_p_ and *I*(0)_st_ are the forward scattering intensities of the protein and standard, respectively. Agreement with the calculated molecular weights of the samples indicates sample quality and monodispersity [81]. Experimental scattering curves were compared with theoretical scattering curves calculated by the program CRYSOL (for *q*_max_<0.3) from the crystal structures of those macromolecules with known atomic structures (CYT-18 NTDs, CYT-18 NTDs+Twort, CYT-18 CTD homology model) [43].

### SAXS *Ab Initio* Shape Reconstructions

*Ab initio* shape reconstructions were done by using DAMMIN (for *q*_max_<8/*R*_g_) and GASBOR, which use simulated annealing methods to build low resolution protein models from dummy atoms or residues, respectively [45],[46]. The program DAMMIN uses dummy atoms packed into a sphere with the beads determined to be either protein or solvent. The final DAMMIN model was obtained by using the DAMAVER program suite to align ten models from independent DAMMIN runs and produce an average model. The latter was further refined by using DAMMIN to produce the final model [82]. GASBOR represents the protein as a chain-like ensemble of dummy residues equal to the number of residues in the protein. The final GASBOR model was chosen as the one with the lowest NSD value after running DAMSEL to compare ten models from independent GASBOR runs [82]. No symmetry was specified for the building of CYT-18* or CYT-18 CTD *ab initio* models, while P2 symmetry was specified for the CYT-18 NTDs models based on prior knowledge from the CYT-18 NTDs crystal structure. DAMMIN and GASBOR produced similar models of CYT-18* with or without P2 symmetry enforced.

### Rigid-Body Modeling

Rigid-body models of CYT-18* by itself and of CYT-18* and the CYT-18 NTDs bound to Twort RNA were built by using the program CORAL [48]. This program employs a simulated annealing method to place high resolution models of individual components in orientations that minimize the discrepancy between the calculated SAXS profile and the experimental SAXS data, with distances between the structured components constrained by randomized dummy residue linkers chosen from a generated library of non-clashing loop structures. To build models of CYT-18*, a homology model of the CYT-18 CTD (amino acids 448–583) was generated by I-TASSER [47], using the *A. nidulans* CTD NMR structure (PDB:2KTL) as a template for modeling [37]. The confidence (C-score) and TM-scores of the CYT-18 CTD homology model, which are indicators of model quality, are high at 0.98 and 0.85, respectively. This CYT-18 CTD model and available high-resolution crystal structures for CYT-18 NTDs+Twort RNA (PDB:2RKJ) were used for rigid-body modeling. The final CORAL models were chosen from among ten independently derived models based on the best fit to the experimental scattering data as indicated by a low χ value [48].

Ensemble optimization analysis to characterize the flexibility of CYT-18*+Twort system was conducted by using the program, EOM [83],[84]. This program generates a random pool of 10,000 structures and creates an optimized ensemble from this pool, such that the average scattering pattern of the ensemble fits the experimental SAXS data. Comparison of the shape of the *R*_g_ and *D*_max_ distributions of the optimized ensemble with those of the random pool provides information about the size and flexibility of the structure, with a broad peak resembling that of the random pool suggesting a flexible, extended structure and a peak narrower than the random pool suggesting a more rigid structure.

### Tyrosyl-Adenylation and TyrRS Assays

Tyrosyl-adenylation assays were done by incubating 100 nM protein in a 50-µl reaction containing 5 mM ATP, 100 mM KCl, 10 mM MgCl_2_, 144 mM Tris-HCl (pH 7.5), 2 mM DTT, 0.1 mg/ml BSA (New England Biolabs), 0.1 unit of yeast inorganic phosphatase (New England Biolabs), and 5 µCi of L-[3,5-^3^H]-tyrosine (53 Ci mmol^−1^; Amersham Biosciences Corp.) [30]. Reactions were initiated by adding protein and incubated at 30°C for 10 min. Reactions were terminated by adding 1 ml of reaction medium and immediately filtering through a nitrocellulose membrane to trap protein bound tyrosyl-adenylate. Radioactivity was measured by Beckman Coulter LS 6500 scintillation counter using Ready Protein scintillation cocktail (Beckman).

Aminoacylation assays were done as described previously with protein concentrations normalized to tyrosyl-adenylation activity [23]. Reactions of 120 µl contained 100 nM protein and 6 µM *E. coli* tRNA^Tyr^ (Sigma-Aldrich) in 100 mM KCl, 15 mM MgCl_2_, 50 mM Tris-HCl (pH 7.5), 5 mM ATP, and 10 mM L-tyrosine (a 1∶10 mixture of L-[3,5-^3^H]-tyrosine and unlabeled L-tyrosine). Reactions were initiated by adding protein and incubated at 30°C. For time courses, 20-µl portions were removed after times ranging from 2 to 60 min, and the reaction was terminated by precipitation with 0.8 ml of a solution containing 10% trichloroacetic acid and 20 mM sodium pyrophosphate. Reactions were filtered through Whatman 3 MM filter paper to collect the precipitates, and the filters were washed three times with 1 ml of a solution containing 5% trichloroacetic acid and 20 mM sodium pyrophosphate followed by 2 ml of 95% ethanol. The filters were then dried and quantified by using a Beckman Coulter LS 6500 scintillation counter as above.

### Equilibrium-Binding Assays

^32^P-labeled RNAs (5 pM; 300 Ci/mmol) were incubated with increasing concentrations of protein in a 50-µl reaction containing 100 mM KCl, 5 mM MgCl_2_, 20 mM Tris-HCl (pH 7.5), 5 mM DTT, 0.1 mg/ml BSA, and 10% glycerol at either 25°C (Figures 4 and 5) or 37°C (Figure S4). Binding reactions were initiated by adding 10 µl protein and terminated after 30 min by filtering 10 µl of the reaction through a nitrocellulose membrane (Amersham Hybond ECL nitrocellulose; GE Healthcare Life Sciences) backed by a nylon membrane (Amersham Hybond-N+; GE Healthcare Life Sciences). The nitrocellulose membrane retains protein-bound RNA and the nylon membrane retains free RNA. The end point (30 min) was chosen after determining that incubations times of 20, 30, or 60 min gave indistinguishable results for all proteins assayed. After application of samples, the membranes were washed three times with 20-µl wash buffer containing 100 mM KCl, 5 mM MgCl_2_, and 20 mM Tris-HCl (pH 7.5), then dried and quantified using a PhosphorImager and the program ImageQuant (GE Healthcare Life Sciences).

### RNA Splicing Assays

Splicing time courses for the Nc mt LSU and Nc *ND1*m introns were done by pre-incubating ^32^P-labeled precursor intron RNA (50 or 200 nM; 0.13–0.4 Ci mmol^−1^) with protein (25 or 100 nM) in a 100-µl reaction containing 100 mM KCl, 5 mM MgCl_2_, 20 mM Tris-HCl (pH 7.5), 1 mM DTT, 0.1 mg/ml BSA, and 10% glycerol for 10 min on ice, followed by 5 min at reaction temperature. Reactions were initiated by adding 1 mM GTP-Mg^2+^. Portions (8 µl) were removed at different times, and the reaction terminated by adding 50 mM EDTA, followed by phenol-CIA extraction and mixing 10 µl of sample with 10 µl of 2× gel loading dye (95% formamide, 0.02% SDS, 0.02% bromophenol blue, 0.01% xylene cyanol, and 1 mM EDTA). End-point splicing assays were done similarly in reaction medium containing 25 or 100 mM KCl for 60 min. Splicing assays comparing wild-type CYT-18 and chimera CYT-18/Sc mtTyrRS proteins were also done with higher concentrations of ^32^P-labeled precursor RNA (200 nM, 0.13–0.4 Ci mmol^−1^) and protein (100 nM or 500 nM dimer) and with 200 nM unlabeled precursor, 500 nM protein, and 500 nM [α-^32^P]GTP (3,000 Ci mmol^−1^; PerkinElmer). In all cases, samples were analyzed by electrophoresis in a denaturing 4% polyacrylamide gel, which was dried and quantified with a PhosphorImager, and data were analyzed by using ImageQuant TL.

## Supporting Information

Data S1 **Excel spreadsheet containing the numerical data and statistical analysis for Figures 2A, 2B, 3A, 3B, 4A–4E, 5A–5C, 7A, 7B, 7E, 7F, S1A–S1D, S2, S4A, S4B, and S5A–S5E.**
(XLSX)

Figure S1 Characterization of the CYT-18* construct. (A) Tyrosyl-adenylation activity of wild-type CYT-18, CYT-18*, CYT-18 NTDs, Sc mtTyrRS, and the Sc NTDs. Assays were done at 30°C, as described in Materials and Methods. The bar graphs show the mean of three experiments, with the error bars indicating the standard deviation. All CYT-18 and *S. cerevisiae* constructs synthesize tyrosyl-adenylate, which remains bound at the active site with a stoichiometry of close to one molecule of tyrosyl-adenylate per protein homodimer, as expected for fully active proteins. (B) Aminoacylation assays of CYT-18 and *S. cerevisiae* protein constructs. The plots show the formation of [^3^H]-Tyr-tRNA^Tyr^ synthesized over time at 30°C (black open circles, wild-type CYT-18; gray open squares, CYT-18*; blue open diamonds, CYT-18 NTDs; orange open triangles, Sc mtTyrRS; green open diamonds, Sc NTDs; black closed circles, no protein control). The assays were done in triplicate with the error bars indicating the standard deviation. (C, D) Splicing activity of wild-type CYT-18 (black open circles) and CYT-18* (gray open squares) with the Nc mt LSU intron at 30°C and 37°C, respectively. Assays were done with 50 nM ^32^P-labeled precursor RNA, 25 nM protein, as described in Materials and Methods. The disappearance of unspliced precursor RNA is plotted over a time period of 60 min. The assays were done in triplicate with the error bars indicating the standard deviation.
(TIF)

Figure S2 **Kratky plots of CYT-18 protein constructs.** The scattering data for (A) CYT-18 NTDs, (B) CYT-18 CTD, and (C) CYT-18* are plotted as q^2^×*I* versus *q*, where *I* is the scattering intensity and *q* is the scattering angle (*q* = 4πsin(θ)/λ). The Kratky plots are normalized to the maximum q^2^×*I* value and show a bell-shape curve with a distinct peak, indicative of a folded globular protein.
(TIF)

Figure S3 ***Ab initio*** **models of CYT-18 proteins built using GASBOR.** CYT-18 NTDs (blue), CTD (red), and CYT-18* (gray) models built by GASBOR, a simulated annealing program which uses a chain-like ensemble of dummy residues [45]. The dummy residue representations are shown above, and the low-resolution SAXS envelopes of the models fit with the CYT-18 NTDs high-resolution structure, the CYT-18 CTD homology model, and the CYT-18* CORAL model using SUPCOMB are shown below. χ alues shown in parentheses indicate the fit of the *ab initio* models to the experimental scattering data. The GASBOR model shown had the lowest NSD among ten calculated models (Table 2).
(TIF)

Figure S4 Size distributions of CYT-18*+Twort optimized ensembles built by EOM. (A) Radius of gyration (*R*_g_) and (B) maximum dimension (*D*_max_) distributions of an optimized ensemble that best describes the experimental scattering data (gray) compared to those of a random pool of conformations (black). The optimized ensemble is selected from the random pool, which consists of 10,000 randomly generated structures. The narrower peak for the size and shape distributions and smaller values of *R*_g_ and *D*_max_ for the optimized ensemble compared to the random pool, suggests that CYT-18*+Twort is a rigid, compact complex.
(TIF)

Figure S5 **Equilibrium-binding assays of CYT-18 deletion mutants to various RNAs at 37°C.** Binding assays of CYT-18 NTDs (blue) and CTD (red) to the (A–C) *N. crassa* mt LSU (Nc mt LSU), *N. crassa ND1*m (Nc *ND1*m), and Twort ribozyme group I intron RNAs; (D) *L. lactis* Ll.LtrB group II intron RNA; and (E) poly(U)_30_. The binding assays were done at 37°C, as described in Materials and Methods. The plots show the fraction of RNA retained on a nitrocellulose membrane as a function of protein concentration. The binding data for the CYT-18 NTDs were fit to hyperbolic curves, while CYT-18 CTD binding data were fit to sigmoidal curves. *K*_d_ or *K*_1/2_ values and Hill coefficients (n) are shown in boxes and are the mean for three experiments with the error bars indicating the standard deviation. The CYT-18 CTD binds similarly to all RNAs tested at 37°C, as it did at 25°C.
(TIF)

Figure S6 **Sequence cloverleaf structure diagrams of tRNA^Tyr^.** The *E. coli* (species 1) tRNA^Tyr^, *S. cerevisiae* mt tRNA^Tyr^, and *N. crassa* mt tRNA^Tyr^ are shown as cloverleafs with the four major identity elements highlighted. The latter are: (1) the N73 nucleotide (purple); (2) the N1–N72 base pair (orange); (3) the anticodon (blue); and (4) the variable arm (green). Modified nucleotides are indicated for each tRNA^Tyr^: D, dihydrouridine; Gm, 2′-O-methylguanosine; i^6^A, N-6-isopentenyladenosine; m^5^C, 5-methylcytidine; m^2,2^G, N2,N2-dimethylguanosine; (m^5^G) 5-methylguanosine. ms^2^i^6^A, N6-(delta 2-isopentenyl)-2-methylthioadenosine; ψ, pseudouridine; Q, queuosine; s^4^U, 4-thiouridine; T, ribothymidine.
(TIF)

Figure S7 **Splicing of the** ***N. crassa*** **mt LSU and** ***ND1*** **m group I introns by CYT-18/Sc mtTyrRS chimeric proteins.** (A–C) Splicing assays of wild-type CYT-18 and chimeric proteins 1 and 2 (Chi.1 and Chi. 2, respectively) with ^32^P-labeled Nc mt LSU group I intron (A and C) or Nc *ND1*m group I intron (B) at two temperatures (25°C or 37°C) and two salt concentrations (25 mM or 100 mM KCl). The splicing reactions were done with 200 nM ^32^P-labeled RNA and 100 nM protein (A and B) or 200 nM ^32^P-labeled RNA and 500 nM protein (C) for 60 min, as described in Materials and Methods. (D) Splicing assays of wild-type CYT-18, Chi.1, and Chi. 2, with unlabeled precursor RNA and [α-^32^P]GTP. Splicing assays were done as above with 500 nM protein, 200 nM unlabeled Nc mt LSU RNA, and 500 nM [α-^32^P]GTP (3,000 Ci mmol^−1^) for 60 min at 25°C. A darker exposure of the autogradiogram is shown below. The chimeric proteins spliced the Nc *ND1*m intron, which is not dependent upon the CYT-18 CTD, but were unable to splice Nc mt LSU intron, which is dependent upon the CYT-18 CTD, under any condition tested. The splicing of the Nc *ND1*m intron by the chimeric proteins in 100 mM KCl decreased with increasing temperature relative to the wild-type protein, likely reflecting loss of CTD interactions that contribute to but are not essential for splicing. Abbreviations: E1–E2, ligated exons; E1-I, 5′ exon+intron; I, excised intron; I-E2, intron+3′ exon; P, precursor RNA.
(TIF)

## Abbreviations

aaRS: aminoacyl-tRNA synthetase
C-tail: C-terminal tail
CTD: C-terminal anticodon-binding domain
*D*_max_: maximum dimension of the particle
mt: mitochondrial
mtTyrRS: mitochondrial tyrosyl-tRNA synthetase
Nc mt LSU intron: *Neurospora crassa* mitochondrial large subunit rRNA-ΔORF intron
Nc *ND1*m intron: *Neurospora crassa* NADH dehydrogenase subunit 1-ΔORF intron
NSD: normalized spatial discrepancy
NTD: N-terminal domain
*P*(*r*): distance distribution function
*q*: momentum transfer
*R*_g_: radius of gyration
SAXS: small angle X-ray scattering
Sc mtTyrRS: *Saccharomyces cerevisiae* mitochondrial tyrosyl-tRNA synthetase
TEV: tobacco etch virus
