# Supplementary figures and images for: Evolution of RNA-Protein Interactions: Non-Specific Binding Led to RNA Splicing Activity of Fungal Mitochondrial Tyrosyl-tRNA Synthetases
Source: PLoS Biol. 2014 Dec 23;12(12):e1002028. doi: 10.1371/journal.pbio.1002028 (PMC4275181; doi:10.1371/journal.pbio.1002028)

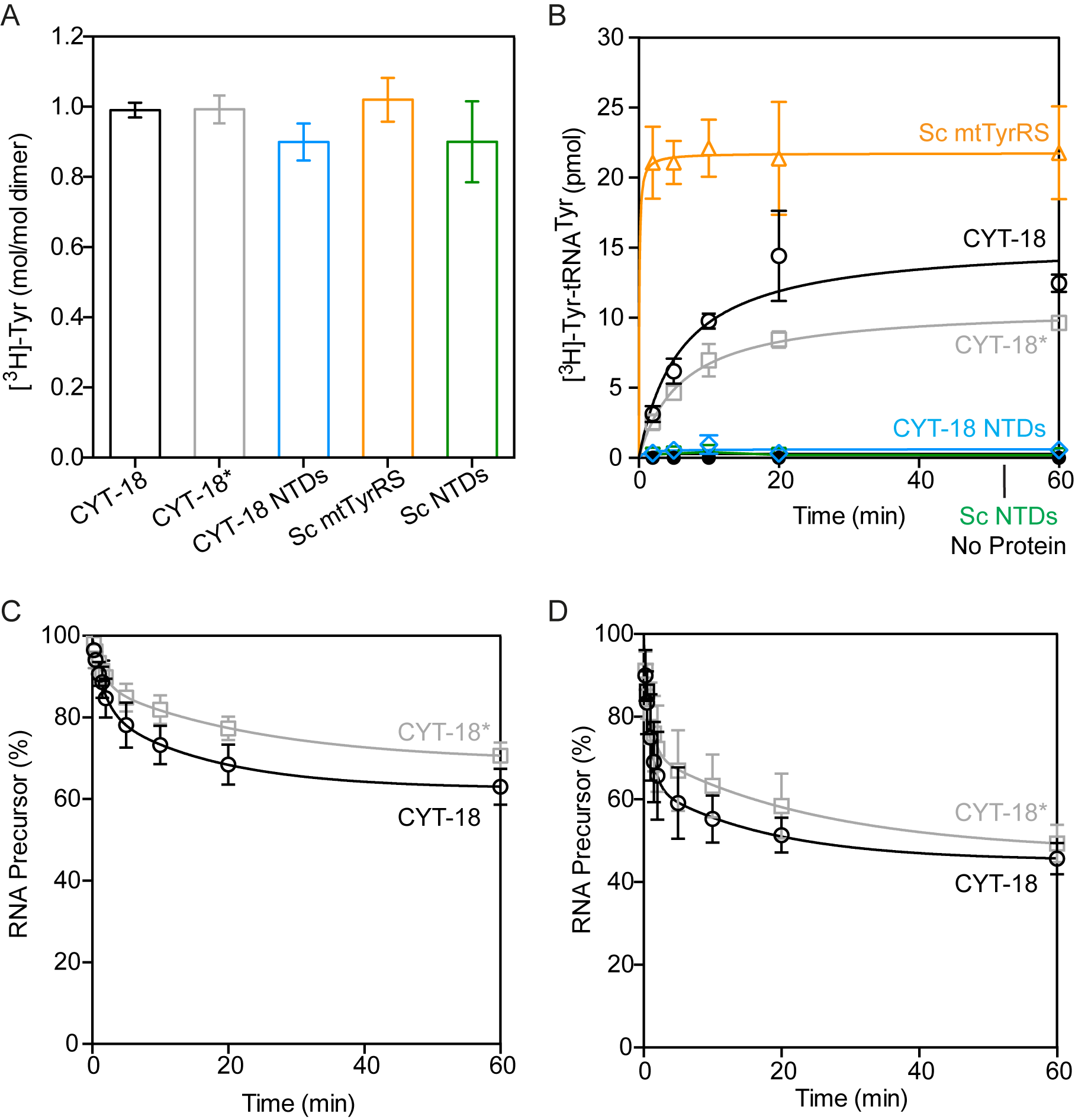

Supplement: Figure S1 — Characterization of the CYT-18* construct. (A) Tyrosyl-adenylation activity of wild-type CYT-18, CYT-18*, CYT-18 NTDs, Sc mtTyrRS, and the Sc NTDs. Assays were done at 30°C, as described in Materials and Methods. The bar graphs show the mean of three experiments, with the error bars indicating the standard deviation. All CYT-18 and S. cerevisiae constructs synthesize tyrosyl-adenylate, which remains bound at the active site with a stoichiometry of close to one molecule of tyrosyl-adenylate per protein homodimer, as expected for fully active proteins. (B) Aminoacylation assays of CYT-18 and S. cerevisiae protein constructs. The plots show the formation of [3H]-Tyr-tRNATyr synthesized over time at 30°C (black open circles, wild-type CYT-18; gray open squares, CYT-18*; blue open diamonds, CYT-18 NTDs; orange open triangles, Sc mtTyrRS; green open diamonds, Sc NTDs; black closed circles, no protein control). The assays were done in triplicate with the error bars indicating the standard deviation. (C, D) Splicing activity of wild-type CYT-18 (black open circles) and CYT-18* (gray open squares) with the Nc mt LSU intron at 30°C and 37°C, respectively. Assays were done with 50 nM 32P-labeled precursor RNA, 25 nM protein, as described in Materials and Methods. The disappearance of unspliced precursor RNA is plotted over a time period of 60 min. The assays were done in triplicate with the error bars indicating the standard deviation. (TIF) [file pbio.1002028.s002.tif]

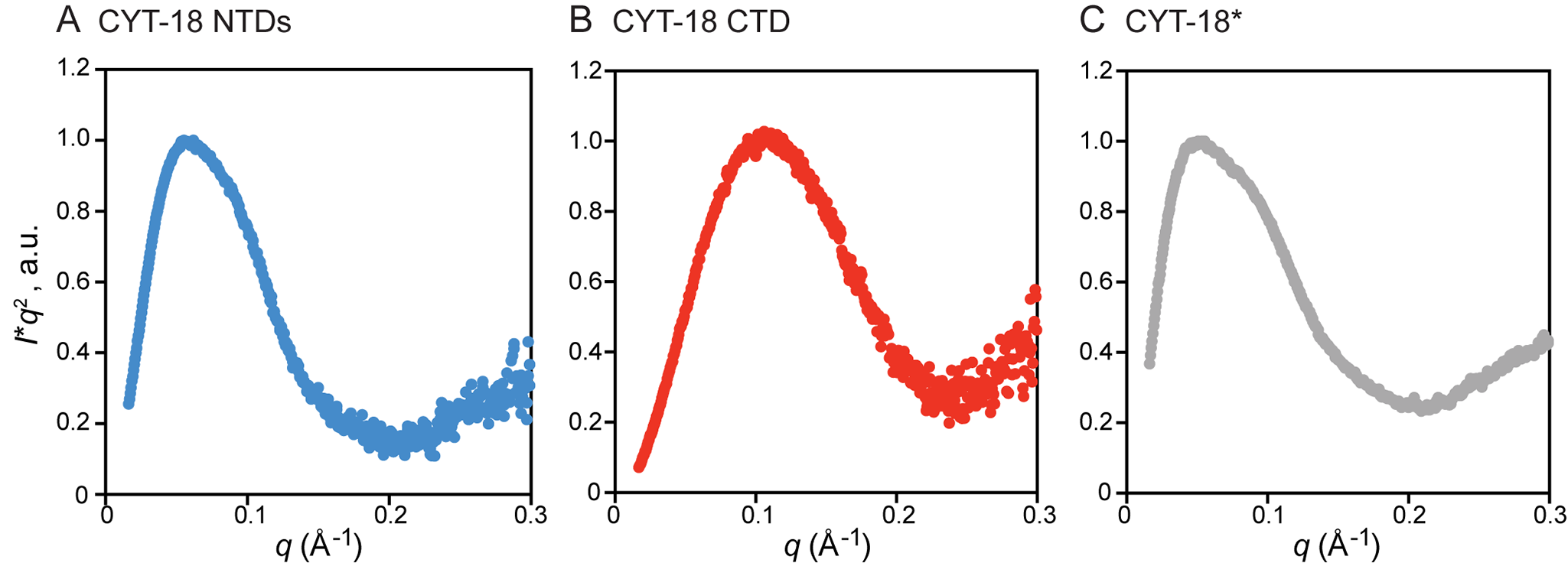

Supplement: Figure S2 — Kratky plots of CYT-18 protein constructs. The scattering data for (A) CYT-18 NTDs, (B) CYT-18 CTD, and (C) CYT-18* are plotted as q2×I versus q, where I is the scattering intensity and q is the scattering angle (q = 4πsin(θ)/λ). The Kratky plots are normalized to the maximum q2×I value and show a bell-shape curve with a distinct peak, indicative of a folded globular protein. (TIF) [file pbio.1002028.s003.tif]

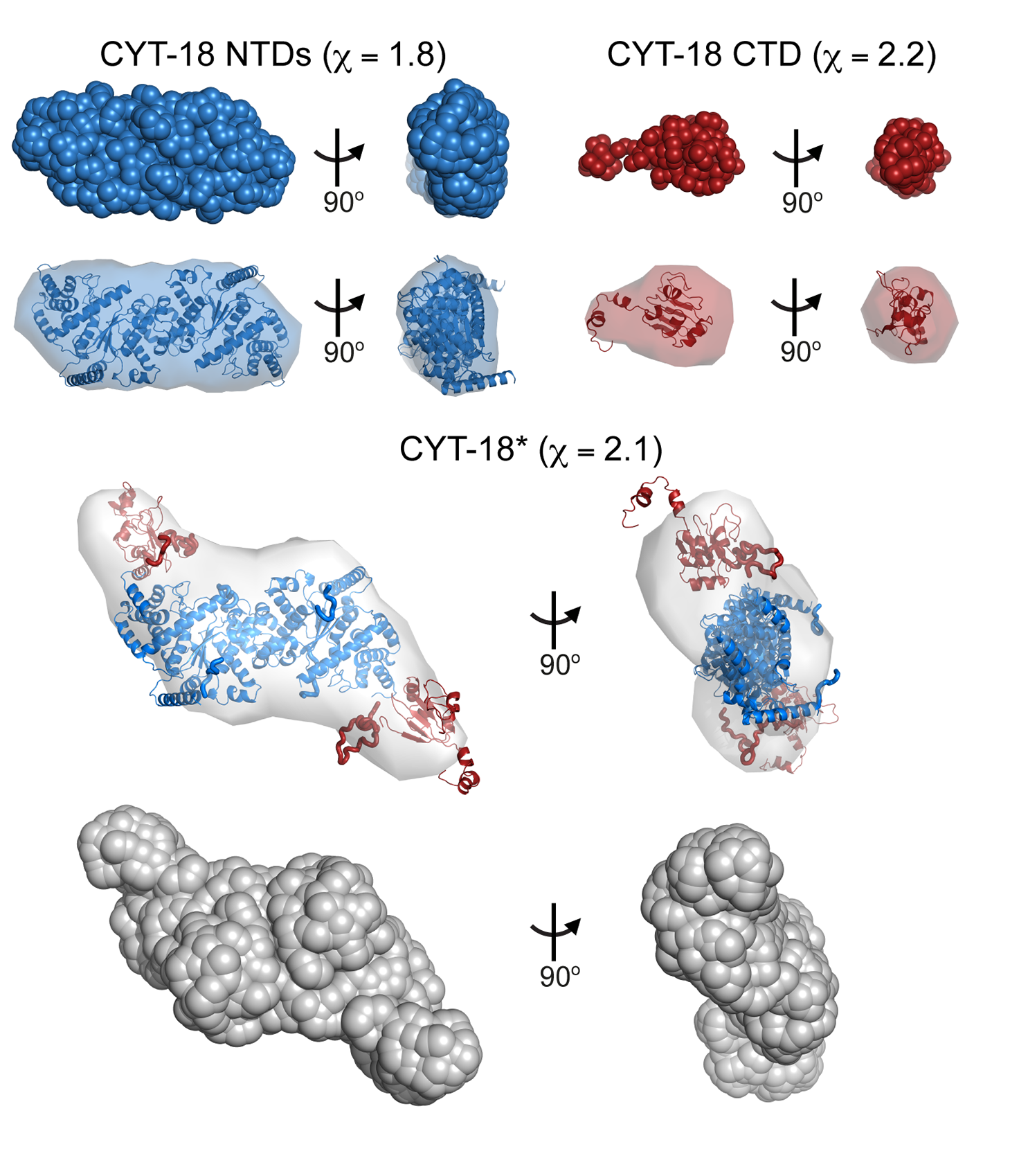

Supplement: Figure S3 — Ab initio models of CYT-18 proteins built using GASBOR. CYT-18 NTDs (blue), CTD (red), and CYT-18* (gray) models built by GASBOR, a simulated annealing program which uses a chain-like ensemble of dummy residues [45]. The dummy residue representations are shown above, and the low-resolution SAXS envelopes of the models fit with the CYT-18 NTDs high-resolution structure, the CYT-18 CTD homology model, and the CYT-18* CORAL model using SUPCOMB are shown below. χ alues shown in parentheses indicate the fit of the ab initio models to the experimental scattering data. The GASBOR model shown had the lowest NSD among ten calculated models (Table 2). (TIF) [file pbio.1002028.s004.tif]

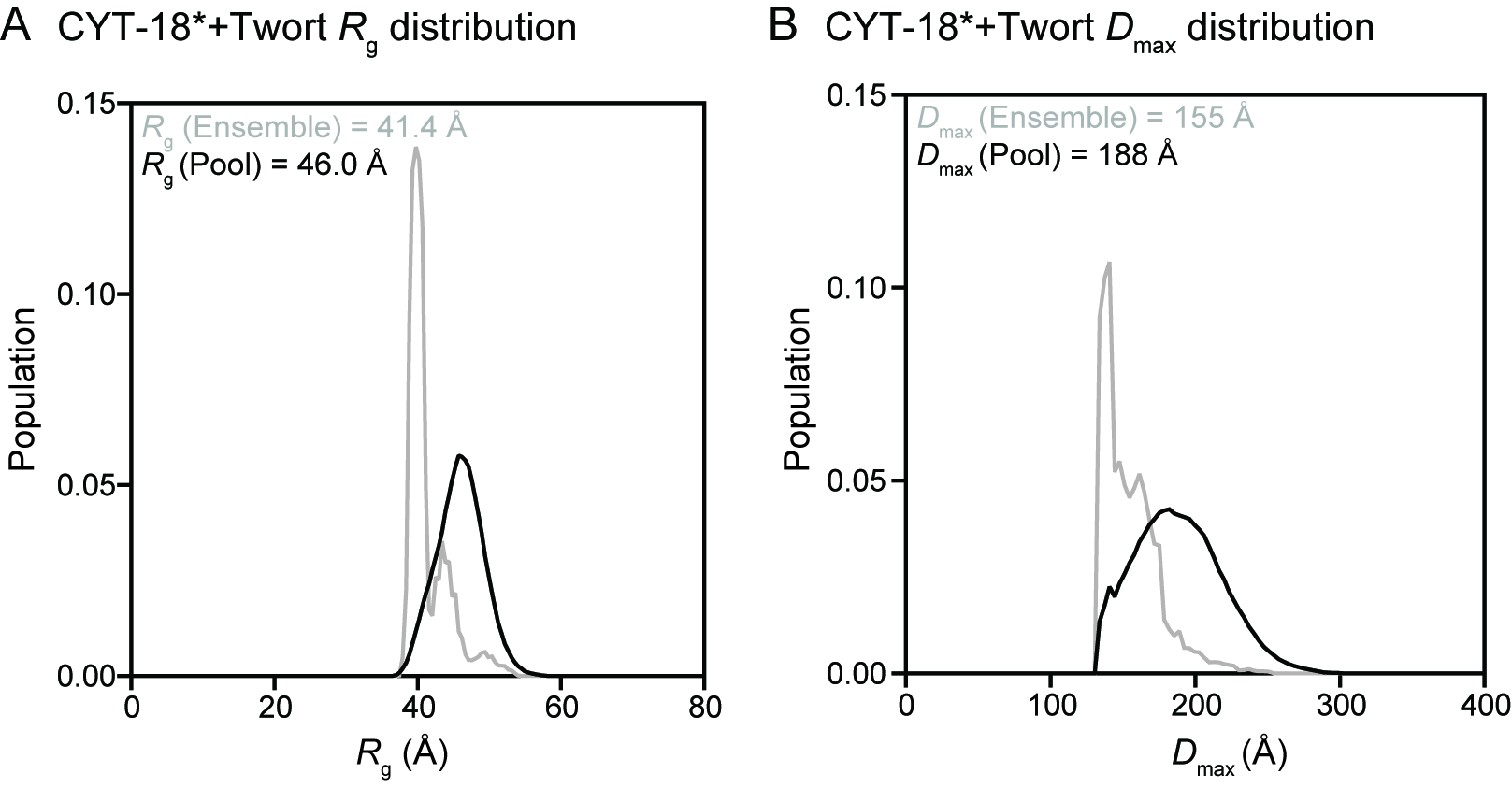

Supplement: Figure S4 — Size distributions of CYT-18*+Twort optimized ensembles built by EOM. (A) Radius of gyration (R g) and (B) maximum dimension (D max) distributions of an optimized ensemble that best describes the experimental scattering data (gray) compared to those of a random pool of conformations (black). The optimized ensemble is selected from the random pool, which consists of 10,000 randomly generated structures. The narrower peak for the size and shape distributions and smaller values of R g and D max for the optimized ensemble compared to the random pool, suggests that CYT-18*+Twort is a rigid, compact complex. (TIF) [file pbio.1002028.s005.tif]

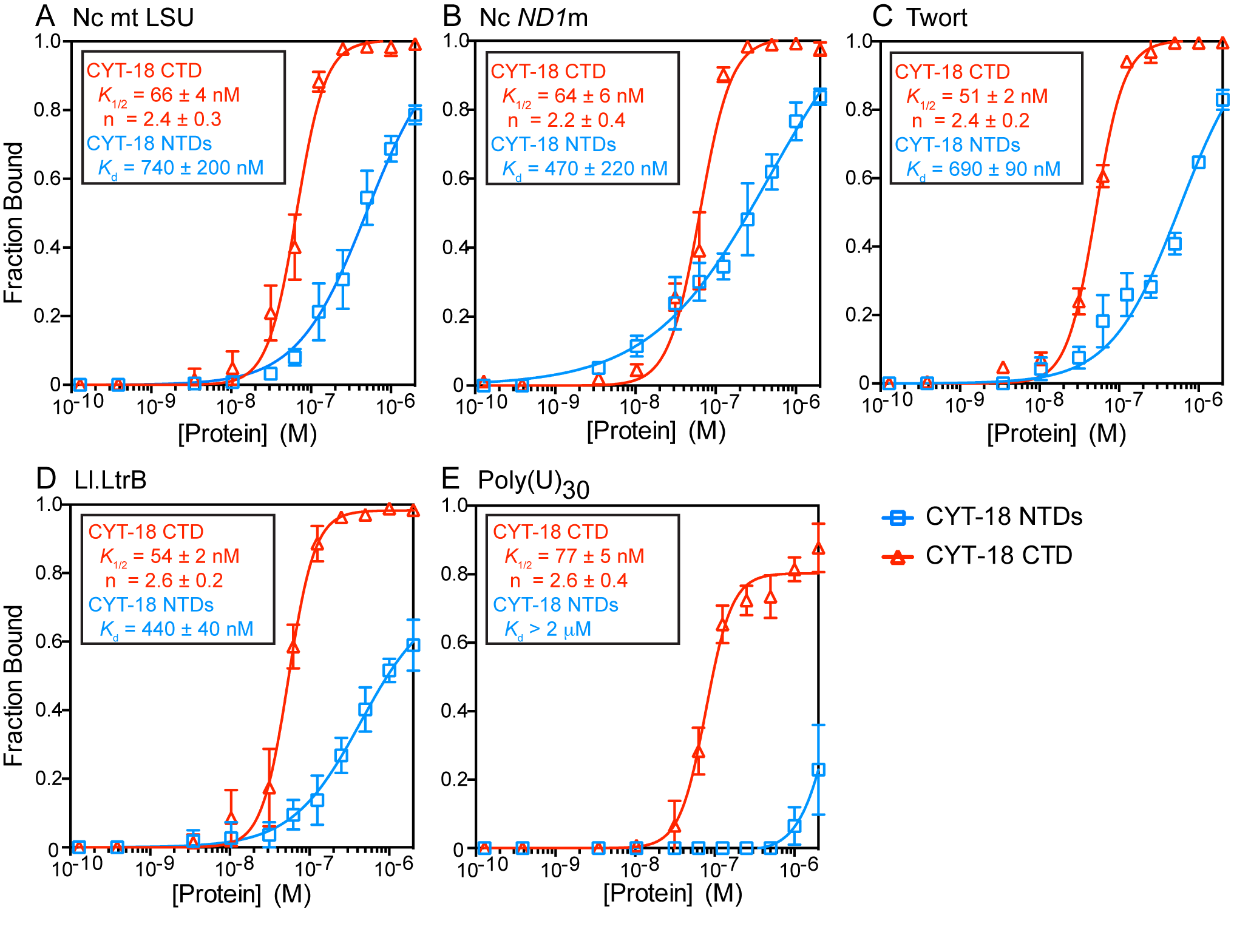

Supplement: Figure S5 — Equilibrium-binding assays of CYT-18 deletion mutants to various RNAs at 37°C. Binding assays of CYT-18 NTDs (blue) and CTD (red) to the (A–C) N. crassa mt LSU (Nc mt LSU), N. crassa ND1m (Nc ND1m), and Twort ribozyme group I intron RNAs; (D) L. lactis Ll.LtrB group II intron RNA; and (E) poly(U)30. The binding assays were done at 37°C, as described in Materials and Methods. The plots show the fraction of RNA retained on a nitrocellulose membrane as a function of protein concentration. The binding data for the CYT-18 NTDs were fit to hyperbolic curves, while CYT-18 CTD binding data were fit to sigmoidal curves. K d or K 1/2 values and Hill coefficients (n) are shown in boxes and are the mean for three experiments with the error bars indicating the standard deviation. The CYT-18 CTD binds similarly to all RNAs tested at 37°C, as it did at 25°C. (TIF) [file pbio.1002028.s006.tif]

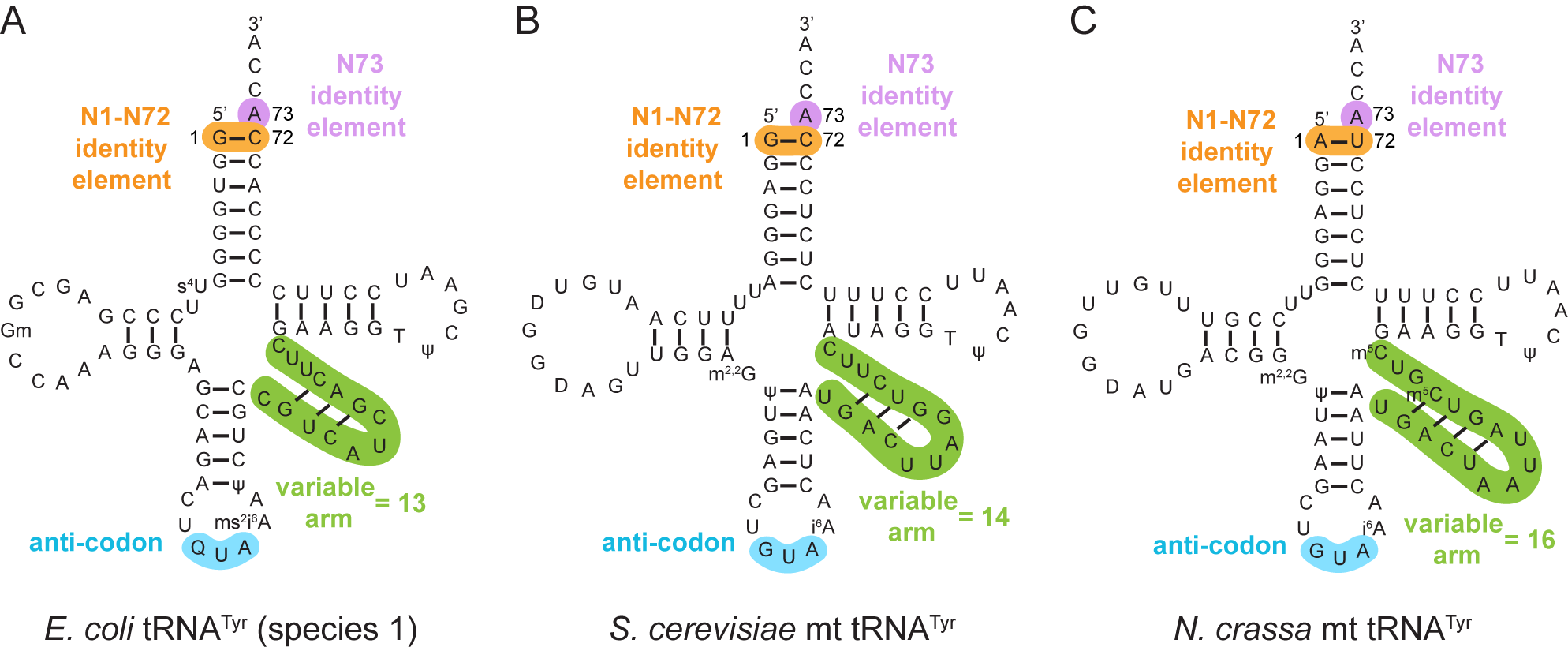

Supplement: Figure S6 — Sequence cloverleaf structure diagrams of tRNATyr. The E. coli (species 1) tRNATyr, S. cerevisiae mt tRNATyr, and N. crassa mt tRNATyr are shown as cloverleafs with the four major identity elements highlighted. The latter are: (1) the N73 nucleotide (purple); (2) the N1–N72 base pair (orange); (3) the anticodon (blue); and (4) the variable arm (green). Modified nucleotides are indicated for each tRNATyr: D, dihydrouridine; Gm, 2′-O-methylguanosine; i6A, N-6-isopentenyladenosine; m5C, 5-methylcytidine; m2,2G, N2,N2-dimethylguanosine; (m5G) 5-methylguanosine. ms2i6A, N6-(delta 2-isopentenyl)-2-methylthioadenosine; ψ, pseudouridine; Q, queuosine; s4U, 4-thiouridine; T, ribothymidine. (TIF) [file pbio.1002028.s007.tif]

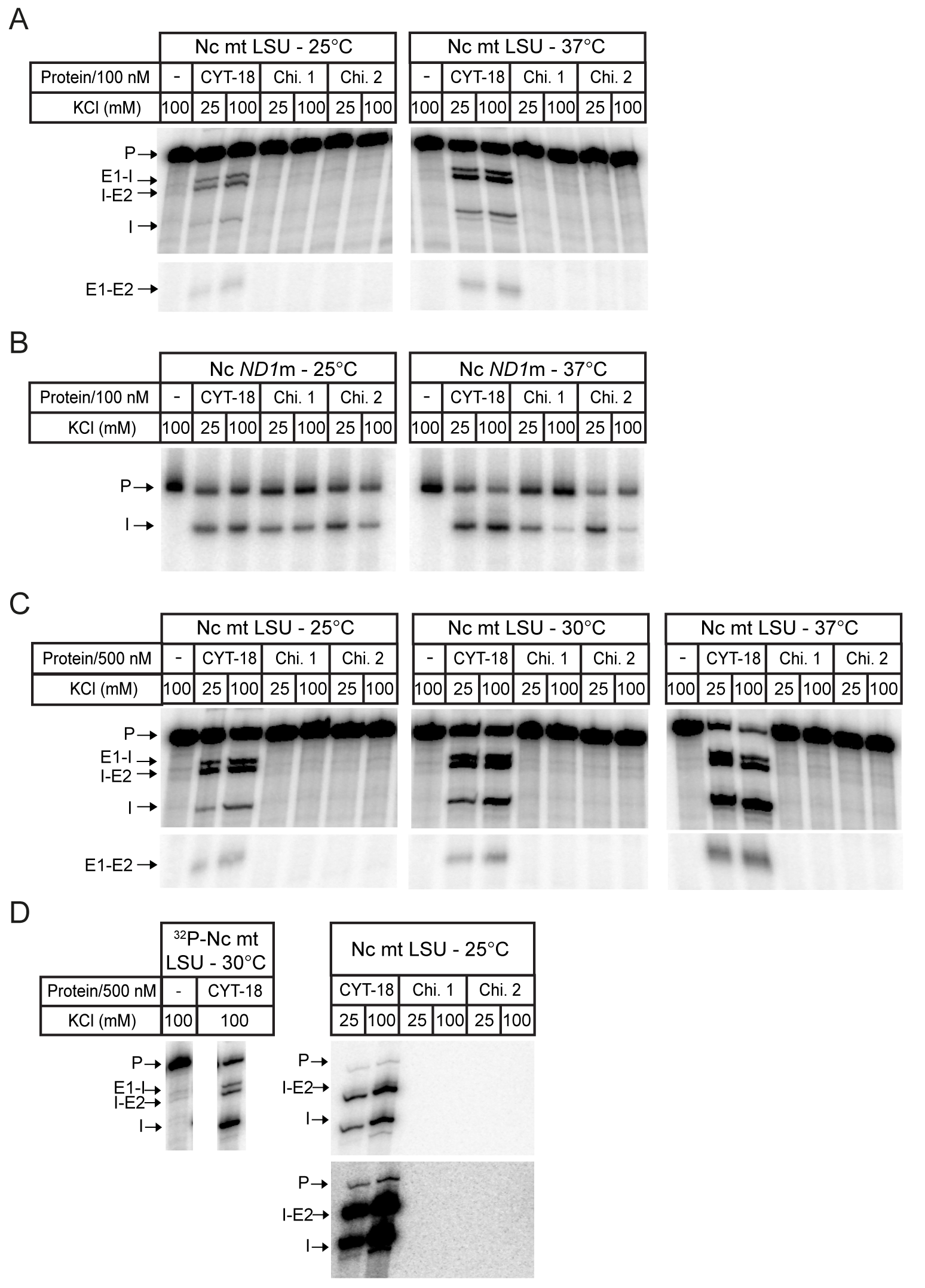

Supplement: Figure S7 — Splicing of the N. crassa mt LSU and ND1 m group I introns by CYT-18/Sc mtTyrRS chimeric proteins. (A–C) Splicing assays of wild-type CYT-18 and chimeric proteins 1 and 2 (Chi.1 and Chi. 2, respectively) with 32P-labeled Nc mt LSU group I intron (A and C) or Nc ND1m group I intron (B) at two temperatures (25°C or 37°C) and two salt concentrations (25 mM or 100 mM KCl). The splicing reactions were done with 200 nM 32P-labeled RNA and 100 nM protein (A and B) or 200 nM 32P-labeled RNA and 500 nM protein (C) for 60 min, as described in Materials and Methods. (D) Splicing assays of wild-type CYT-18, Chi.1, and Chi. 2, with unlabeled precursor RNA and [α-32P]GTP. Splicing assays were done as above with 500 nM protein, 200 nM unlabeled Nc mt LSU RNA, and 500 nM [α-32P]GTP (3,000 Ci mmol−1) for 60 min at 25°C. A darker exposure of the autogradiogram is shown below. The chimeric proteins spliced the Nc ND1m intron, which is not dependent upon the CYT-18 CTD, but were unable to splice Nc mt LSU intron, which is dependent upon the CYT-18 CTD, under any condition tested. The splicing of the Nc ND1m intron by the chimeric proteins in 100 mM KCl decreased with increasing temperature relative to the wild-type protein, likely reflecting loss of CTD interactions that contribute to but are not essential for splicing. Abbreviations: E1–E2, ligated exons; E1-I, 5′ exon+intron; I, excised intron; I-E2, intron+3′ exon; P, precursor RNA. (TIF) [file pbio.1002028.s008.tif]
